# A novel MAP kinase‐interacting protein MoSmi1 regulates development and pathogenicity in *Magnaporthe oryzae*

**DOI:** 10.1111/mpp.13493

**Published:** 2024-07-21

**Authors:** Yu Wang, Xinyue Cui, Junlian Xiao, Xiaoru Kang, Jinmei Hu, Zhicheng Huang, Na Li, Chuyu Yang, Yuemin Pan, Shulin Zhang

**Affiliations:** ^1^ Department of Plant Pathology, College of Plant Protection Anhui Agricultural University Hefei China; ^2^ Anhui Province Key Laboratory of Crop Integrated Pest Management Anhui Agricultural University Hefei China; ^3^ State Key Laboratory for Managing Biotic and Chemical Threats to the Quality and Safety of Agro‐Products, College of Life Sciences Zhejiang University Hangzhou China

**Keywords:** appressorium formation, cell wall integrity, pathogenicity, regulation, rice blast fungus

## Abstract

The cell wall is the first barrier against external adversity and plays roles in maintaining normal physiological functions of fungi. Previously, we reported a nucleosome assembly protein, MoNap1, in *Magnaporthe oryzae* that plays a role in cell wall integrity (CWI), stress response, and pathogenicity. Moreover, MoNap1 negatively regulates the expression of *MoSMI1* encoded by *MGG_03970*. Here, we demonstrated that deletion of *MoSMI1* resulted in a significant defect in appressorium function, CWI, cell morphology, and pathogenicity. Further investigation revealed that MoSmi1 interacted with MoOsm1 and MoMps1 and affected the phosphorylation levels of MoOsm1, MoMps1, and MoPmk1, suggesting that MoSmi1 regulates biological functions by mediating mitogen‐activated protein kinase (MAPK) signalling pathway in *M. oryzae*. In addition, transcriptome data revealed that MoSmi1 regulates many infection‐related processes in *M. oryzae*, such as membrane‐related pathway and oxidation reduction process. In conclusion, our study demonstrated that MoSmi1 regulates CWI by mediating the MAPK pathway to affect development and pathogenicity of *M. oryzae*.

## Graphical abstract

MoSmi1 regulates biological functions by mediating the mitogen‐activated protein kinase (MAPK) signalling pathway in *Magnaporthe oryzae*.

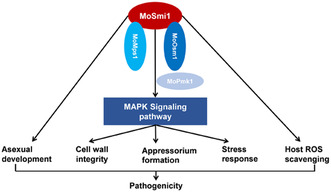

## 1 INTRODUCTION

Rice blast, caused by *Magnaporthe oryzae*, is one of the most destructive fungal diseases that decrease rice yield and seriously threaten food security (Dean et al., 2012). *M. oryzae* infection begins with a three‐celled conidium. The conidium attaches to the host leaf surface and develops into a germ tube within 2 h. Subsequently, a domed‐shaped structure called the appressorium differentiates from the tip of the germ tube (Hamer et al., 1988; Hamer & Talbot, 1998; Howard & Valent, 1996). With the accumulation of high concentrations of glycerol, the appressorium generates sufficient turgor pressure to form an infection peg and penetrates the host cell (Howard et al., 1991). Subsequently, typical necrotic spots of rice blast appear on plant surfaces after about 5 days. Finally, the invasive hyphae spread within plant cells, resulting in typical lesions, and the secondary conidia spread the disease to adjacent plants (Gupta et al., 2021). In the process by which the rice blast fungus infects the host, numerous signal transduction pathways receive and transduce extracellular signals to regulate growth, development, and pathogenicity of *M. oryzae* (Li et al., 2012).

Mitogen‐activated protein kinase (MAPK)‐mediated pathways have been shown to regulate appressorium development, appressorium‐mediated penetration, cell wall integrity (CWI) and response to stress in *M. oryzae* (Cai et al., 2022; Tucker & Talbot, 2001). Appressorium‐mediated penetration is important for pathogenicity of *M. oryzae*. During appressorium formation, G protein‐coupled receptors (GPCRs) recognize hydrophobic surface signals and activate the G protein signalling pathway, which controls crosstalk between the cAMP‐PKA and Pmk1 MAPK signalling pathways in *M. oryzae* (Ebbole, 2007; Kou & Naqvi, 2016; Li et al., 2012; McDonough & Rodriguez, 2011). The CWI MAPK cascade is composed of MoMck1 (Bck1 homologue), MoMkk1 (Mkk1/2 homologue), and MoMps1 (Slt2/Mpk1 homologue). To maintain cell morphology, MoMck1 delivers signals to MAPK kinase, MoMkk1, which in turn activates the MAPK MoMps1 through protein phosphorylation. MoMps1 phosphorylates downstream transcription factors to regulate the nuclear expression of genes involved in cell wall biosynthesis and cell cycle progression (Jeon et al., 2008; Xu et al., 1998; Yin et al., 2016). Cell wall remodelling is important for maintaining fungal growth and development. In *M. oryzae*, *MoMCK1* regulates cell wall remodelling and resists plant defences (Jeon et al., 2008). The Osm1 MAPK pathway consists of MoSsk2, MoPbs2, MoOsm1, and an adaptor protein MoMst50, which play essential roles in response to hyperosmotic stress (Dixon et al., 1999; Li et al., 2017).

We previously reported a nucleosome assembly protein, MoNap1, that regulates appressorium formation, response to cell wall stresses, cytoplasmic division, and virulence (Zhang, Wang, et al., 2022). Based on transcriptome data previously, we screened for differentially expressed genes (DEGs) between the wild‐type Guy11 and Δ*Monap1* mutant and identified *MGG_03970*. Bioinformatics analysis revealed that the protein product encoded by *MGG_03970* is homologous to Smi1. Smi1, also known as Knr4, is an intrinsically disordered protein (IDP) conserved in many fungi (Martin‐Yken et al., 2016). In *Saccharomyces cerevisiae*, Smi1 acts as a hub that physically interacts with the key components of two pathways: Rho GTPase‐protein kinase C‐MAP kinase in the CWI pathway and calcineurin phosphatase in the calcium‐calcineurin pathway (Dagkessamanskaia, Durand, et al., 2010; Dagkessamanskaia, El Azzouzi, et al., 2010; Martin‐Yken et al., 2016). Knr4 has diverse biological functions, including cell cycle progression, CWI, morphogenesis, and response to heat and cell wall stress, by regulating associated transcriptional programmes (Lagorce et al., 2003; Martin‐Yken et al., 2002; Penacho et al., 2012). In *S. cerevisiae*, Knr4 participates in Cln3‐Cdc28‐dependent gene transcription with Bck2 at the G1/S transition (Kuravi et al., 2011; Martin‐Yken et al., 2002). Additionally, loss of Knr4 results in the disordered function of at least two cell cycle checkpoints: the morphogenesis checkpoint, which combines cell division with bud growth, and the mechanism controlling daughter cell size during cytokinesis (Dagkessamanskaia, Durand, et al., 2010; Dagkessamanskaia, El Azzouzi, et al., 2010; Harrison et al., 2001; Miyakawa & Mizunuma, 2007; Mizunuma et al., 2001). In the human pathogen *Candida albicans*, Smi1 expression is induced in the pathogenic hyphal cells; the *smi1*Δ*/smi1*Δ mutant shows reduced cell wall β‐glucan synthesis and biofilm formation and reduced biofilm‐associated fluconazole resistance, which suggests a positive effect on the CWI pathway (Harcus et al., 2004; Nett et al., 2011). In *Fusarium asiaticum*, FaSmi1 is a key protein required for the vegetative development, asexual reproduction, deoxynivalenol (DON) production and virulence (Zhang, Chen, et al., 2022). In addition, *KNR4* contributes to a significantly increased release of polysaccharides and mannoproteins into the culture medium, which is of special interest in oenological fermentation processes (Gonzalez‐Ramos et al., 2008).

Although there is some evidence that Smi1 is involved in the CWI pathway, how Smi1 regulates MAPK cascades and the pathogenicity of the blast fungus remains unclear. Here, we evaluated the functions of MoSmi1 and revealed that MoSmi1 regulates vegetative growth, conidiation, cell morphology, appressorium formation, host reactive oxygen species (ROS) scavenging, CWI, and pathogenicity. In addition, MoSmi1 interacts with MoOsm1 and MoMps1, which are key components of the Osm1 and CWI MAPK pathways, respectively. Phosphorylation levels of MoOsm1, MoMps1, and MoPmk1 were increased in Δ*Mosmi1*. Our study reveals that MoSmi1 affects the biological functions of *M. oryzae* by regulating MAPK signalling cascades.

## 2 RESULTS

### 2.1 Identification and analysis of MoSmi1 in *M. oryzae*

Through RNA sequencing (RNA‐seq) analysis, we previously found that the gene *MGG_03970* was upregulated in the Δ*Monap1* mutant (Zhang, Chen, et al., 2022; Zhang, Wang, et al., 2022). To verify the reliability of transcriptome data, we assessed the transcription levels of *MGG_03970* in Guy11 and Δ*Monap1* mutant using reverse transcription‐quantitative PCR (RT‐qPCR). The results showed that the transcription level of *MGG_03970* was upregulated approximately 1.6 times in the Δ*Monap1* mutant compared with that in the wild‐type strain Guy11 (Figure S1). This gene encodes a 571‐amino acid (aa) protein with the Smi1_Knr4 domain. Therefore, we named *MGG_03970* as *MoSMI1* in *M. oryzae*. Phylogenetic analysis revealed that Smi1 is conserved in the fungal kingdom, and MoSmi1 has the highest homology with Smi1 of *Colletotrichum graminicola* (Figure S2a). Domain prediction analysis revealed that Smi1 contains a Smi1_Knr4 domain in various fungi (Figure S2b). We predicted three intrinsically disordered regions (IDRs) in the amino acid sequence of MoSmi1 (Figure S2c). In addition, MoSmi1 is closely related to proteins from other fungi. The results from multiple sequence alignment indicated that MoSmi1 shares 58% amino acid identity to that of *Fusarium graminearum*, 52% to *Trichoderma reesei*, 56% to *Neurospora crassa*, and 33% to *Schizosaccharomyces pombe* (Figure S2d). Taken together, these results revealed that the Smi1 proteins are conserved in fungi.

### 2.2 Subcellular localization of MoSmi1 at different developmental stages in *M. oryzae*

To determine the subcellular localization of MoSmi1 at different developmental stages in *M. oryzae*, a MoSmi1‐green fluorescence protein (GFP) construct under the control of its native promoter was transformed into the wild‐type strain Guy11. GFP signals were examined in mycelia, conidia, appressoria, and invasive hypha using confocal microscopy. We observed that MoSmi1‐GFP was mainly distributed throughout the cytoplasm in all tested stages and vacuoles at the appressorium formation stage. In addition, punctate green fluorescence was observed in the conidia (Figure S3A). We further stained appressoria with 7‐amino‐4‐chloromethylcoumarin (CMAC) and verified that MoSmi1‐GFP was also localized in vacuoles at the appressorium formation stages (Figure S3B). These results suggest that MoSmi1 plays an important role in all developmental and plant infection processes of *M. oryzae*.

### 2.3 MoSmi1 functions in vegetative growth, conidiation and morphogenesis in *M. oryzae*

To investigate the biological functions of *MoSMI1* in *M. oryzae*, we generated the *MoSMI1* deletion mutant in the wild‐type strain Guy11 background using a homologous recombination strategy (Figure S4a). The putative *MoSMI1* deletion mutant transformants were verified by PCR, RT‐PCR and Southern blotting (Figure S4b–d). We named the *MoSMI1* deletion mutant as Δ*Mosmi1*. In addition, the genetic complementation strain for the Δ*Mosmi1* mutant was generated by introducing the *MoSMI1* gene with its native promoter into the Δ*Mosmi1* mutant (#2), namely, Δ*Mosmi1/MoSMI1* (HB strain), and verified by RT‐PCR analysis (Figure S4d). The wild‐type strain Guy11, Δ*Mosmi1* mutant, and the complemented strain Δ*Mosmi1/MoSMI1* were used for phenotypic analyses.

**FIGURE 1:**
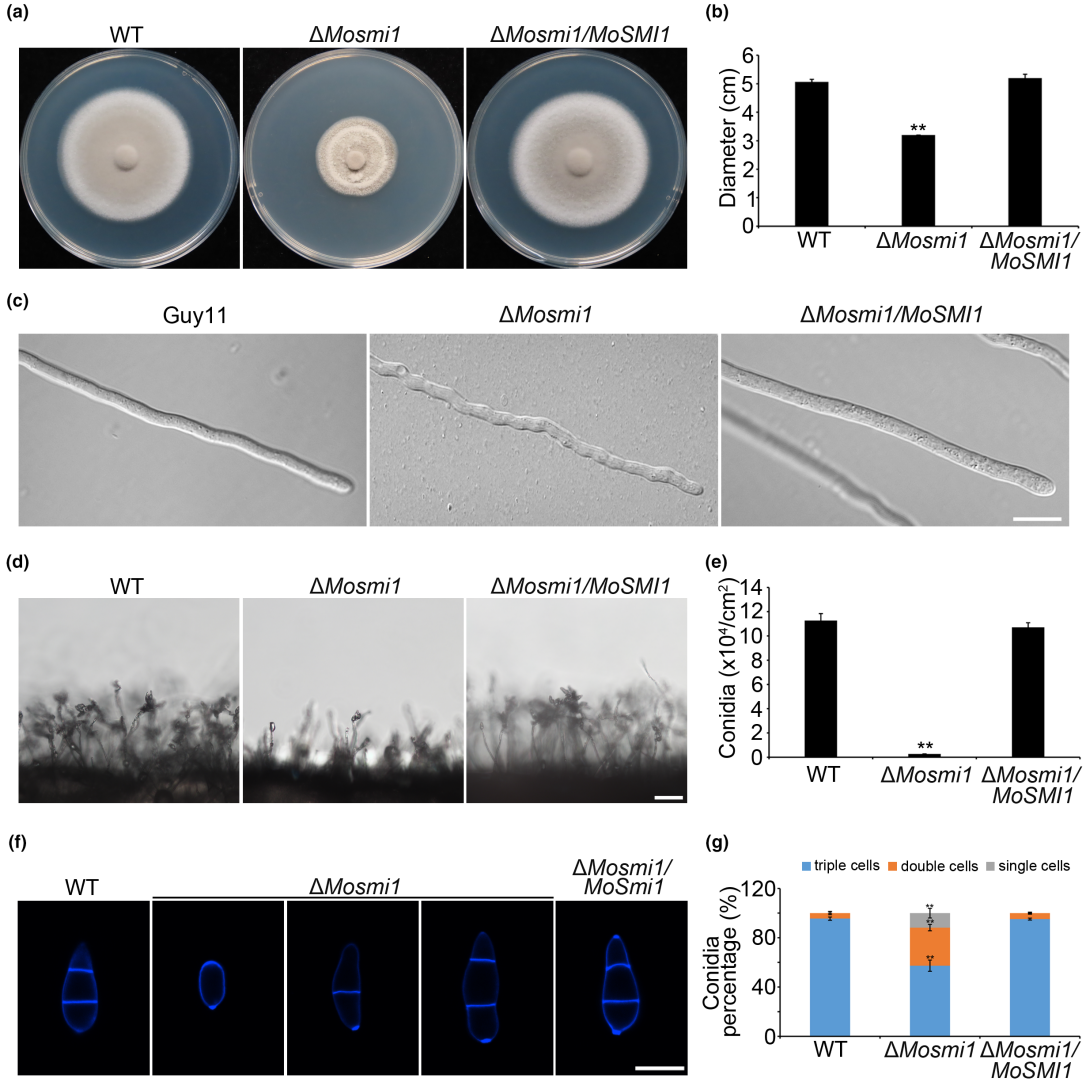
*MoSMI1* is important for vegetative growth, mycelial morphology, conidiation, and conidial morphology of *Magnaporthe oryzae*. (a) Colonies of the wild‐type strain Guy11 (WT), Δ*Mosmi1* mutant, and the complemented strain Δ*Mosmi1/MoSMI1* on complete medium (CM) plates were observed and captured after 7 days at 28°C. (b) Colony diameters were measured and statistically analysed. For each strain, three independent biological experiments were performed with four replicates each time. Error bars represent *SD* and asterisks indicate significant differences between the WT strain Guy11 and ∆*Mosmi1* mutant estimated using Student's *t* test (**p < 0.01). (c) The hyphal morphology of all tested strains. All the tested strains were cultured in liquid CM for 48 h and photographed under an inverted fluorescent microscope. Bar, 20 μm. (d) All the strains were incubated on an artificial hydrophobic surface for 24 h at 28°C. Conidia and conidiophore formation were observed and photographed using an inverted fluorescent microscope. Bar, 50 μm. (e) Statistical analysis of the conidiation of all tested strains. For each strain, three independent biological experiments with four replicates were performed each time. Error bars represent *SD* and asterisks indicate significant differences between the wild‐type strain Guy11, Δ*Mosmi1* mutant estimated using Student's *t* test (**p < 0.01). (f) Conidial morphology of the tested strains. Conidia collected from the WT strain Guy11, Δ*Mosmi1* mutant and complemented strain Δ*Mosmi1/MoSMI1* were stained with calcofluor white (CFW) and photographed under an inverted fluorescent microscope. Bar, 20 μm. (g) Proportion of each conidial type. One hundred conidia were counted for each strain and three experiments were performed. Error bars represent *SD* and asterisks indicate significant differences (*p < 0.05).

To investigate the contribution of *MoSMI1* in vegetative growth of *M. oryzae*, we cultured the wild‐type strain Guy11, Δ*Mosmi1* mutant, and the complemented strain Δ*Mosmi1/MoSMI1* on complete medium (CM) plates for 7 days at 28°C in darkness. The colony diameter of the Δ*Mosmi1* mutant was significantly smaller than that of the wild‐type strain Guy11 and the complemented strain Δ*Mosmi1/MoSMI1* (Figure 1a,b). In addition, we assessed the morphology of the vegetative hyphae in all tested strains. Results from this assay indicated that the vegetative hyphae of the Δ*Mosmi1* mutant showed a curved morphology, suggesting *MoSMI1* is important for the maintenance of polarized growth (Figure 1c). Given that conidia are the primary infection propagules of *M. oryzae*, we also examined whether *MoSMI1* deletion affects sporulation capacity and conidial development. As shown in Figure 1d, the number of spores on the conidiophores of the Δ*Mosmi1* mutant was significantly lower than that on the conidiophores of the wild‐type strain Guy11, and the complemented strain Δ*Mosmi1/MoSMI1*. We counted the number of conidia and found that conidiation of theΔ*Mosmi1* mutant was significantly decreased (Figure 1e). Moreover, calcofluor white (CFW) staining assay indicated that approximately 50% of the Δ*Mosmi1* mutant conidia were one‐ or two‐celled (Figure 1f,g). Taken together, these results suggest that *MoSMI1* is involved in vegetative growth, conidiation, and conidial development of *M. oryzae*.

### 2.4 Deletion of *MoSMI1* disrupts microtubule structure and nuclear distribution in hyphae

**FIGURE 2:**
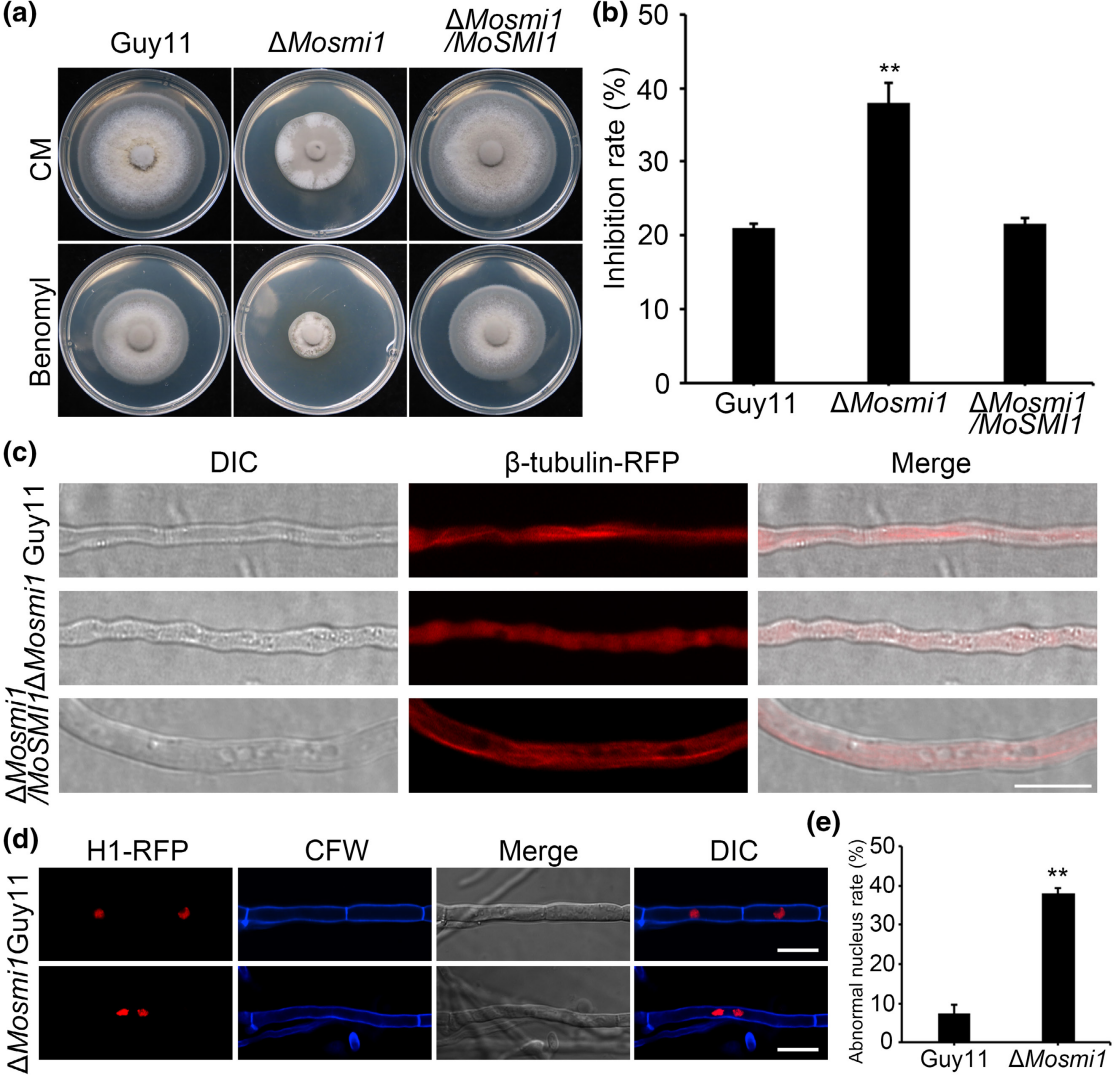
*MoSMI1* is required for the organization of microtubule and cytoplasmic division in *Magnaporthe oryzae*. (a) Colonies of the wild‐type strain Guy11, Δ*Mosmi1* mutant, and complemented strain Δ*Mosmi1/MoSMI1* were cultured in complete medium (CM) plates containing 15 μg/mL benomyl in darkness at 28°C for 7 days. (b) Statistical analysis of the relative inhibition rate (%) of the tested strains. For each strain, three independent biological experiments with four replicates were performed each time. Error bars represent *SD*, and asterisks above the columns indicate significant differences between the wild‐type strain Guy11, Δ*Mosmi1* mutant estimated by Student's *t* test (**p < 0.01). (c) Subcellular localization of β‐tubulin‐RFP in the wild‐type strain Guy11, and the Δ*Mosmi1* mutant, and the complemented strain Δ*Mosmi1/MoSMI1* in vegetative hyphae stage. Bar, 10 μm. (d) Subcellular localization of H1‐RFP in the wild‐type strain Guy11 and Δ*Mosmi1* mutant in vegetative hyphae. Bar, 10 μm. Cell wall was visualized using calcofluor white (CFW). The fluorescence signals were observed using a laser scanning confocal microscope. (e) Statistical analysis of the proportion of abnormal number of cell nuclei in a single cell. At least 100 hyphal cells were counted in each strain. Three experiments were performed. Error bars represent *SD* and asterisks indicate significant differences (**p < 0.01).

As shown in the above result, deletion of *MoSMI1* affected the polarized growth of vegetative hyphae. We inferred that *MoSMI1* may play a role in regulation of microtubule dynamics. To assess the role of *MoSMI1* in microtubule, we examined the sensitivity of all tested strains to the microtubule inhibitor benomyl (Hoyt et al., 1991). As shown in Figure 2a,b, the relative inhibition rate of the Δ*Mosmi1* mutant was higher than that of wild‐type strain Guy11, and the complemented strain Δ*Mosmi1/MoSMI1*, suggesting that MoSmi1 affects microtubule function. Furthermore, we expressed a β‐tubulin‐red fluorescence protein (RFP) fusion protein in the wild‐type strain Guy11, Δ*Mosmi1* mutant and the complemented strain Δ*Mosmi1/MoSMI1* background. Microscopic examination showed that β‐tubulin‐RFP of the wild‐type strain Guy11 and the complemented strain Δ*Mosmi1/MoSMI1* background were parallel to their growth axes and formed a linear structure in the cytoplasm. In contrast, in the Δ*Mosmi1* mutant background, the linear structure of β‐tubulin‐RFP was disrupted and the RFP signal exhibited a dispersed state (Figure 2c). In addition, we treated the wild‐type strain Guy11 expressing β‐tubulin‐RFP with 0.4 μg/mL benomyl for 6 h and found that the linear RFP signal gradually dispersed (Figure S5), which was similar to the Δ*Mosmi1* mutant, further indicating that *MoSMI1* affects microtubule formation. Microtubules are important for normal cell cycle progression (Hoyt et al., 1991). Therefore, we expressed Histone 1 (H1‐RFP) in the wild‐type strain Guy11 and Δ*Mosmi1* mutant and stained hyphae using CFW to determine whether *MoSMI1* deletion affects nuclear distribution. We found that in wild‐type Guy11, there was only one nucleus in most hyphal cells (92%), However, the number of nuclei was abnormal (no or more than one) in 38% hyphal cells of the Δ*Mosmi1* mutant, suggesting that MoSmi1 is involved in regulating cytokinesis of *M. oryzae* (Figure 2d,e). Overall, these results indicated that MoSmi1 regulates the microtubule structure and nuclear distribution in *M. oryzae*.

### 2.5 MoSmi1 is required for pathogenicity of *M. oryzae*

**FIGURE 3:**
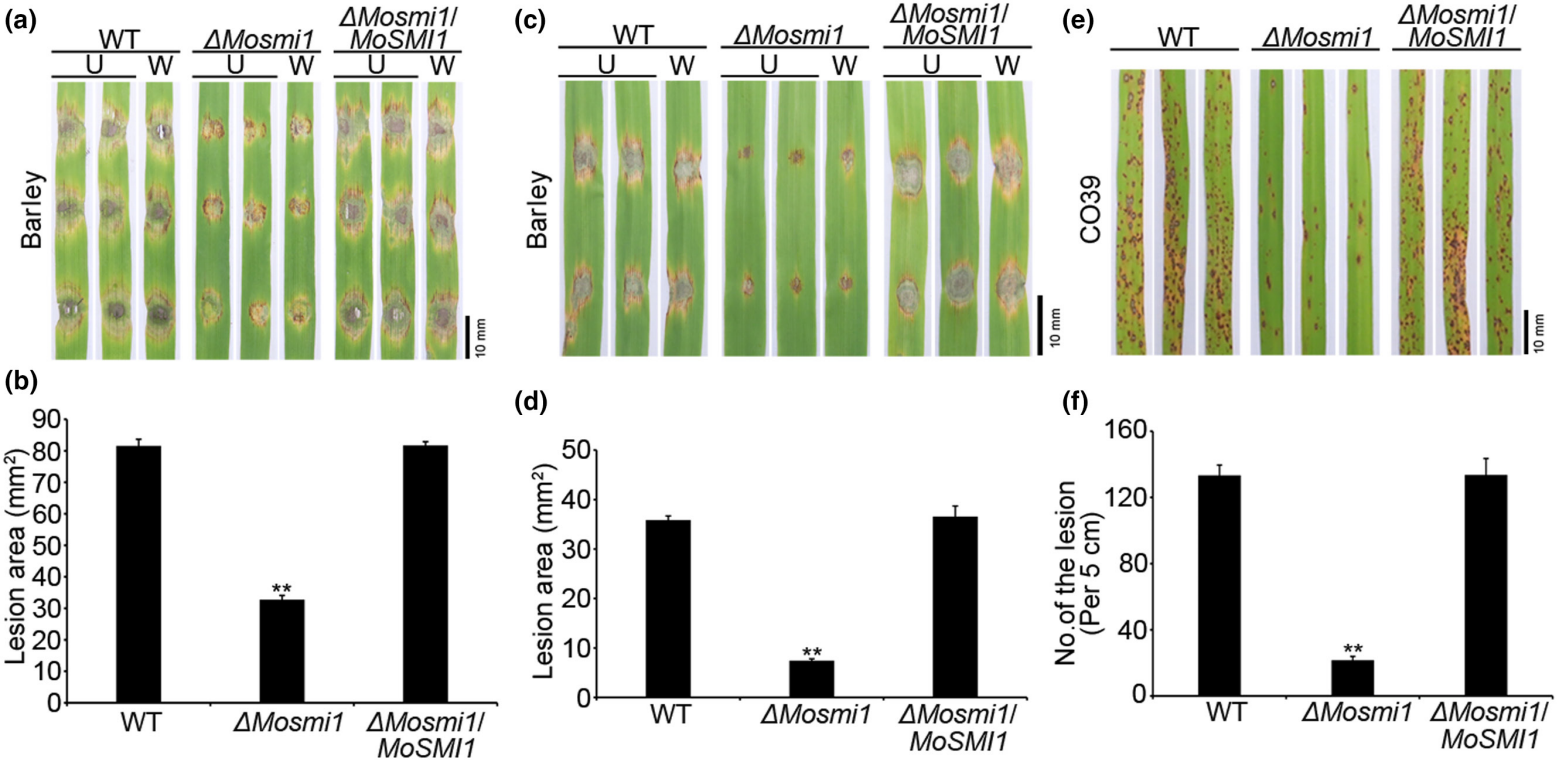
*MoSMI1* is required for pathogenicity of *Magnaporthe oryzae*. (a) Pathogenicity on barley leaves. Mycelial agar plugs of all tested strains were inoculated on 7‐day‐old barley leaves and photographed at 5 days post‐inoculation (dpi). U, unwounded (intact) leaf; W, wounded leaf. Bar, 10 mm. (b) Statistical analysis of the lesion area of all tested strains on barley leaves using ImageJ software. Three experiments were performed. Error bars represent *SD* and asterisks indicate significant differences (**p < 0.01). (c) Pathogenicity on barley leaves. Conidial suspensions (5 × 10^4^ conidia/mL) of all tested strains were dropped on 7‐day‐old barley leaves and photographed at 5 dpi. Bar, 10 mm. (d) Statistical analysis of the lesion area of all tested strains on barley leaves using ImageJ software. Three experiments were performed. Error bars represent *SD* and asterisks indicate significant differences (**p < 0.01). (e) Pathogenicity on rice seedlings. Conidial suspensions (5 × 10^4^ conidia/mL in a 0.2% wt/vol gelatin solution) from each tested strain were sprayed onto 14‐day‐old rice seedlings and photographed at 5 dpi. Bar, 10 mm. (f) Lesion numbers were counted within a 5 cm length of leaf from each strain, and a minimum of three leaves were assessed for each strain. Three experiments were performed. Error bars represent *SD* and asterisks indicate significant differences (**p < 0.01).

To determine whether deletion of *MoSMI1* affects the pathogenicity of *M. oryzae*, we tested the virulence of the wild‐type strain Guy11, Δ*Mosmi1* mutant, and the complemented strain Δ*Mosmi1/MoSMI1* on the susceptible barley cv. Golden Promise and susceptible rice seedlings (*Oryzae sativa* ‘CO39’). First, mycelial plugs or conidial suspensions (5 × 10^4^ conidia/mL) of all tested strains were inoculated on detached barley leaves. At 5 days post‐inoculation (dpi), the Δ*Mosmi1* mutant caused more restricted lesions in both intact and wounded leaves than the wild‐type strain Guy11 and the complemented strain Δ*Mosmi1/MoSMI1* (Figure 3a–d). Furthermore, conidial suspensions (5 × 10^4^ conidia/mL) of all tested strains were sprayed onto susceptible CO39 rice seedlings. At 7 dpi, the wild‐type strain Guy11 and the complemented strain Δ*Mosmi1/MoSMI1* produced typical lesions on rice leaves, whereas the Δ*Mosmi1* mutant produced smaller lesions (Figure 3e,f). Based on these results, we conclude that *MoSMI1* plays an important role in the pathogenicity of *M. oryzae*.

### 2.6 MoSmi1 affects appressorium formation, invasive hyphal expansion and host ROS scavenging

**FIGURE 4:**
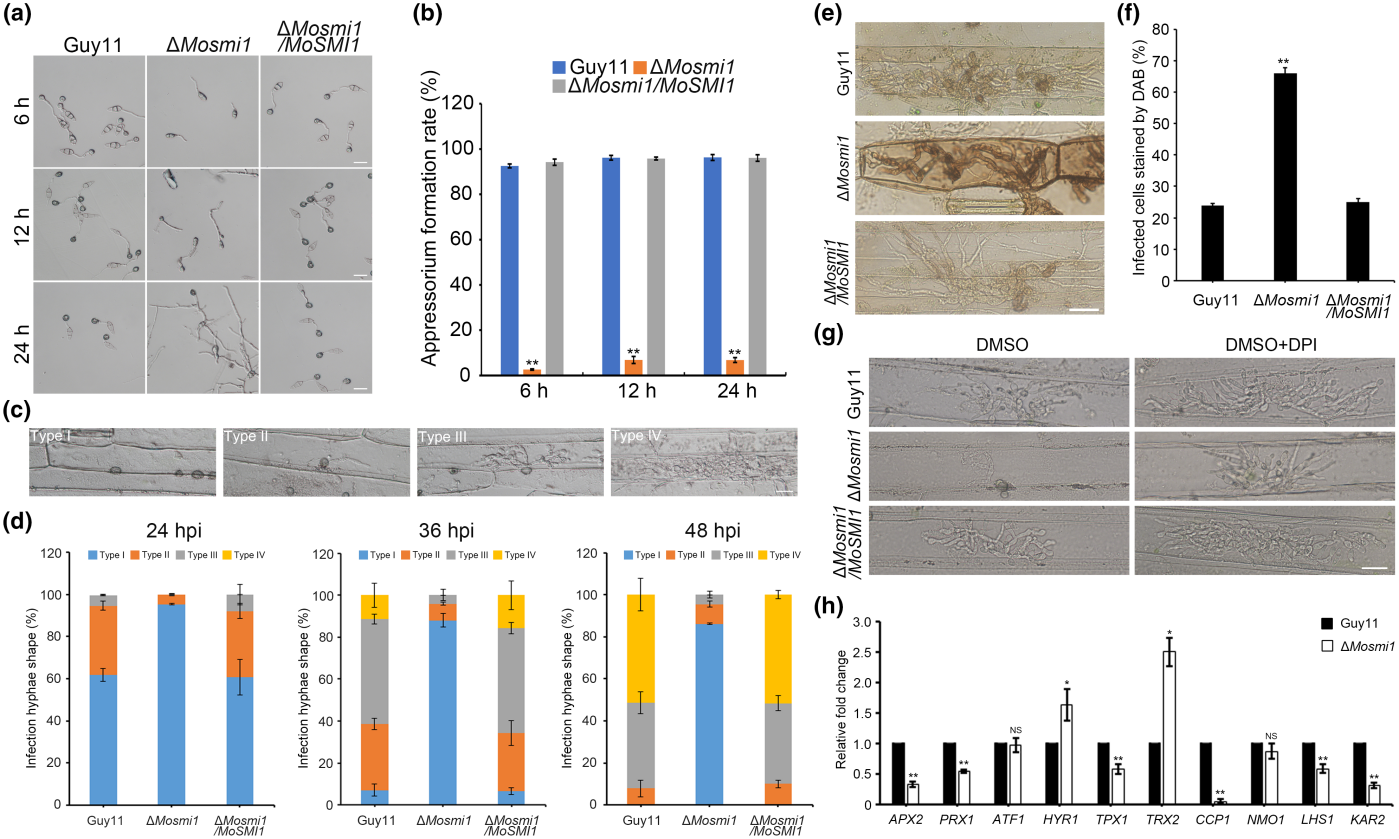
MoSmi1 affects appressorium formation, invasive hyphae (IH) expansion, and host reactive oxygen species (ROS) scavenging. (a) Conidial suspensions (5 × 10^4^ conidia/mL) of all the tested strains were inoculated on an artificial hydrophobic surface and viewed at 6, 12, and 24 h post‐inoculation (hpi). Bar, 20 μm. (b) Statistical analysis of appressorium formation rate (%) of all tested strains. A minimum of 100 conidia were observed and counted in each strain. Three experiments were performed. Error bars represent *SD* and asterisks indicate significant differences (**p < 0.01). (c) Conidial suspensions (5 × 10^4^ conidia/mL) of all tested strains were dropped on the back of barley leaves, and barley epidermal cells were observed at 24, 36, and 48 hpi. Type I, only penetration peg without invasive hypha; Type II, only one single invasive hypha without branches; Type III, more than one branch but restricted to one host cell; Type IV, more than one branch and extended to neighbouring host cells. Bar, 20 μm. (d) Statistical analysis of four types of IH. At least 100 penetration sites were counted for each strain. Three experiments were performed. Error bars represent *SD.* (e) Conidial suspensions of all tested strains were inoculated onto barley leaves for 30 h and stained with 3,3′‐diaminobenzidine (DAB) solution. Bar, 25 μm. (f) Statistical analysis of the proportion of infected cells stained by DAB. For each strain, at least 100 invading cells were observed and the number of stained cells was counted. Error bars represent *SD* and asterisks indicate significant differences (**p < 0.01). (g) Barley leaves were inoculated with conidial suspensions of all tested strains treated with diphenyleneiodonium (DPI), and IH growth was observed at 30 hpi. Dimethyl sulphoxide (DMSO) treatment was a control that was used to dissolve DPI. Bar, 25 μm. (h) Relative expression of 10 ROS detoxification‐related genes in the wild‐type Guy11 and Δ*Mosmi1* mutant. The *β‐tubulin* gene (*MGG_00604*) was used as the reference gene. Three independent biological experiments with three replicates were performed. Error bars represent *SD* and asterisk represents significant differences (*p < 0.05, **p < 0.01, NS, *p* > 0.05).

To explore the cause of the pathogenicity defect in the Δ*Mosmi1* mutant, we first determined whether deletion of *MoSMI1* affects appressorium formation in *M. oryzae*. Conidial suspensions (5 × 10^4^ conidia/mL) of all the tested strains were inoculated on hydrophobic coverslips, and appressorium formation was observed at different time points (6, 12, and 24 h). We observed less than 10% of the Δ*Mosmi1* mutant conidia formed appressoria, in contrast to more than 90% of the wild‐type strain Guy11 and the complemented strain Δ*Mosmi1/MoSMI1* conidia at all time points (Figure 4a,b). This result indicated that *MoSMI1* is important for appressorium formation in *M. oryzae*. Furthermore, we performed a penetration assay using the wild‐type strain Guy11, Δ*Mosmi1* mutant, and the complemented strain Δ*Mosmi1/MoSMI1* on the barley epidermis. Invasive hyphae (IH) were classified into four types: type I (no hyphal penetration), type II (IH with one branch), type III (IH with at least two branches, but not extending to the neighbouring cells), and type IV (IH that has numerous branches and extends to the neighbouring cells). At 24 h post‐inoculation (hpi), the wild‐type strain Guy11 and the complemented strain Δ*Mosmi1*/*MoSMI1* formed approximately 35% IH as type II and approximately 5% IH as type III, whereas the Δ*Mosmi1* mutant produced more than 90% as type I. At 36 hpi, more than 85% IH were type I in the Δ*Mosmi1* mutant, compared with approximately 5% type I, 30% type II, 50% type III, and 15% type IV in the wild‐type strain Guy11 and the complemented strain Δ*Mosmi1/MoSMI1*. Even if at 48 hpi, the Δ*Mosmi1* mutant formed more than 80% type I IH. However, more than 50% IH were type IV and type I no longer existed in the wild‐type strain Guy11 and the complemented strain Δ*Mosmi1/MoSMI1* (Figure 4c,d). These data indicate that MoSmi1 regulates IH expansion. Taken together, these results demonstrate that *MoSMI1* deletion delays appressorium formation and restricts IH expansion.

Given the deficient invasive growth of Δ*Mosmi1*, we speculated that host ROS inhibit the extension of IH during infection. A 3,3′‐diaminobenzidine (DAB) staining experiment was performed to detect ROS accumulation in barley leaf cells. At 30 hpi, only approximately 25% plant cells infected with the wild‐type strain Guy11 and the complemented strain Δ*Mosmi1/MoSMI1* were stained, whereas approximately 65% of plant cells infected by the Δ*Mosmi1* mutant were stained with DAB (Figure 4e,f). These results suggest that ROS accumulation increased in barley leaf cells infected with the Δ*Mosmi1* mutant. We hypothesized that this was due to the limited ROS scavenging ability of the Δ*Mosmi1* mutant. Furthermore, diphenyleneiodonium (DPI) was used to treat barley epidermal cells. We found that at 30 hpi when treated with 0.5 μM DPI, the invasive growth was greatly enhanced compared with the control dimethyl sulfoxide (DMSO) treatment (Figure 4g). In addition, we measured the expression level of 10 ROS detoxification‐related genes (Guo et al., 2010; Huang et al., 2011; Ren et al., 2022; Yi et al., 2009) in the wild‐type strain Guy11 and the Δ*Mosmi1* mutant. As shown in Figure 4h, *APX2*, *PRX1*, *TPX1*, *CCP1*, *LHS1*, and *KAR2* were significantly downregulated in Δ*Mosmi1* compared with the wild‐type strain Guy11. The relative expression levels of *HYR1* and *TRX2* were slightly upregulated, and there was no significant difference in *ATF1* and *NMO1* in the Δ*Mosmi1* mutant compared with the wild‐type strain Guy11. Taken together, our data indicate that MoSmi1 is involved in scavenging host ROS and that the defective pathogenicity of Δ*Mosmi1* was partly due to the accumulation of ROS.

### 2.7 MoSmi1 affects the organization of the septin ring in *M. oryzae*

**FIGURE 5:**
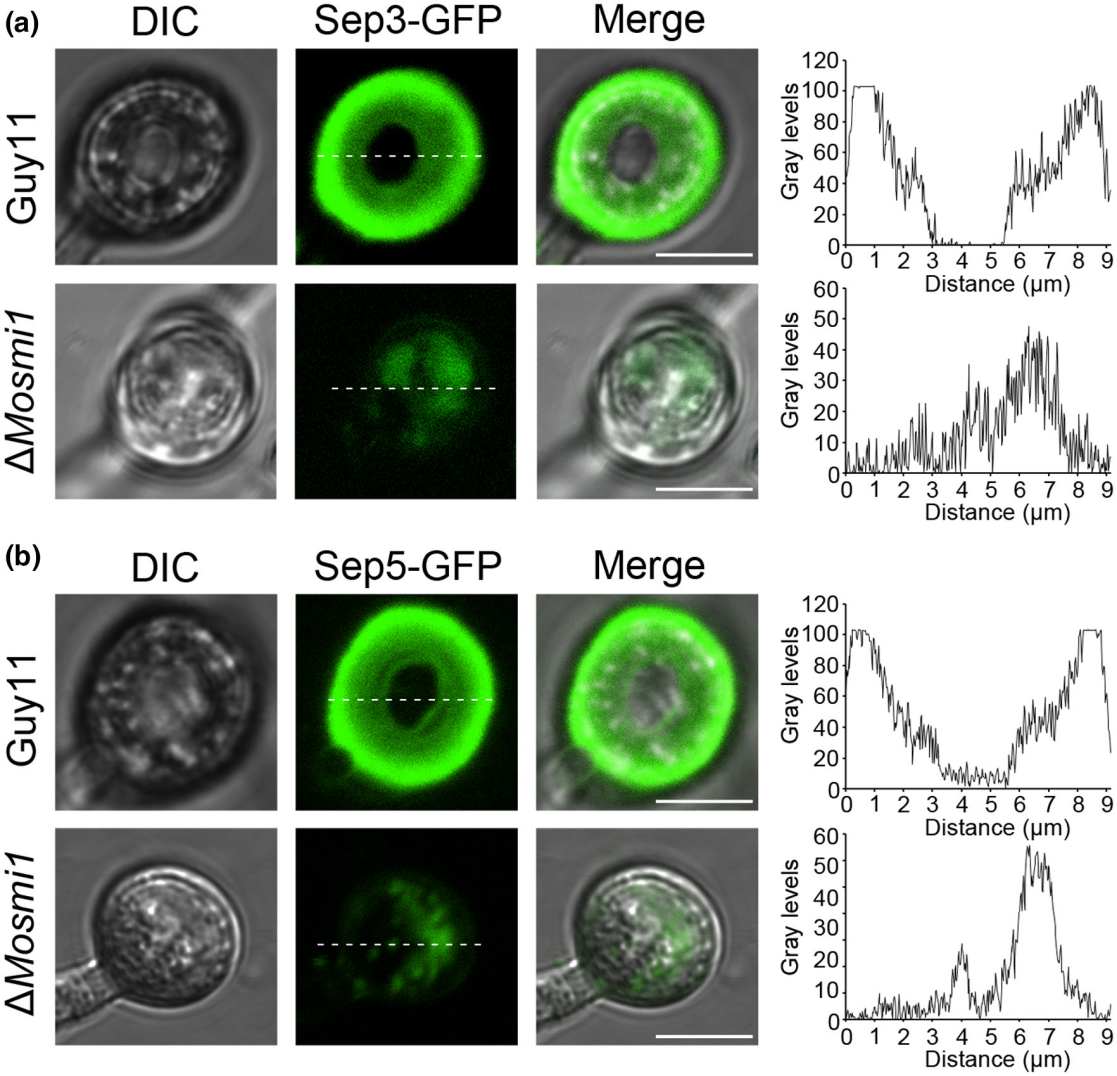
*MoSMI1* affects septin ring formation in *Magnaporthe oryzae*. (a, b) The conidial suspensions (5 × 10^4^ conidia/mL) of the wild‐type Guy11 and Δ*Mosmi1* mutant expressing Sep3‐GFP or Sep5‐GFP were inoculated on an artificial hydrophobic surface and the appressoria were observed at 24 h post‐inoculation under a laser scanning confocal microscope. The distribution of the fluorescence signal in a transverse section (indicated by the white dotted line) was analysed using ImageJ software. Bar, 5 μm.

A normal appressorium is key for pathogen penetration, which relies on the recruitment and organization of septin‐dependent cytoskeletal components (Li et al., 2021). Previous study demonstrated that septin proteins bind phosphatidylinositol phosphates at the appressorium pore membrane to assemble into a ring, promoting the formation of a penetration peg that is required for host infection by *M. oryzae* (Dagdas et al., 2012). Considering there is delayed appressorium formation and restricted penetration by the Δ*Mosmi1* mutant, we speculated that the septin ring structure may be abnormal in the Δ*Mosmi1* mutant. To test this, we expressed Sep3‐GFP and Sep5‐GFP in the wild‐type strain Guy11 and Δ*Mosmi1* mutant. At 24 hpi, both Sep3‐GFP and Sep5‐GFP exhibited a ring structure in the appressorium centre in the wild‐type Guy11 background, whereas the GFP fluorescence signal was disordered in the Δ*Mosmi1* mutant (Figure 5a,b). In summary, our results suggest that MoSmi1 is involved in septin ring formation in *M. oryzae*.

### 2.8 MoSmi1 is required for cell wall integrity and stress response

**FIGURE 6:**
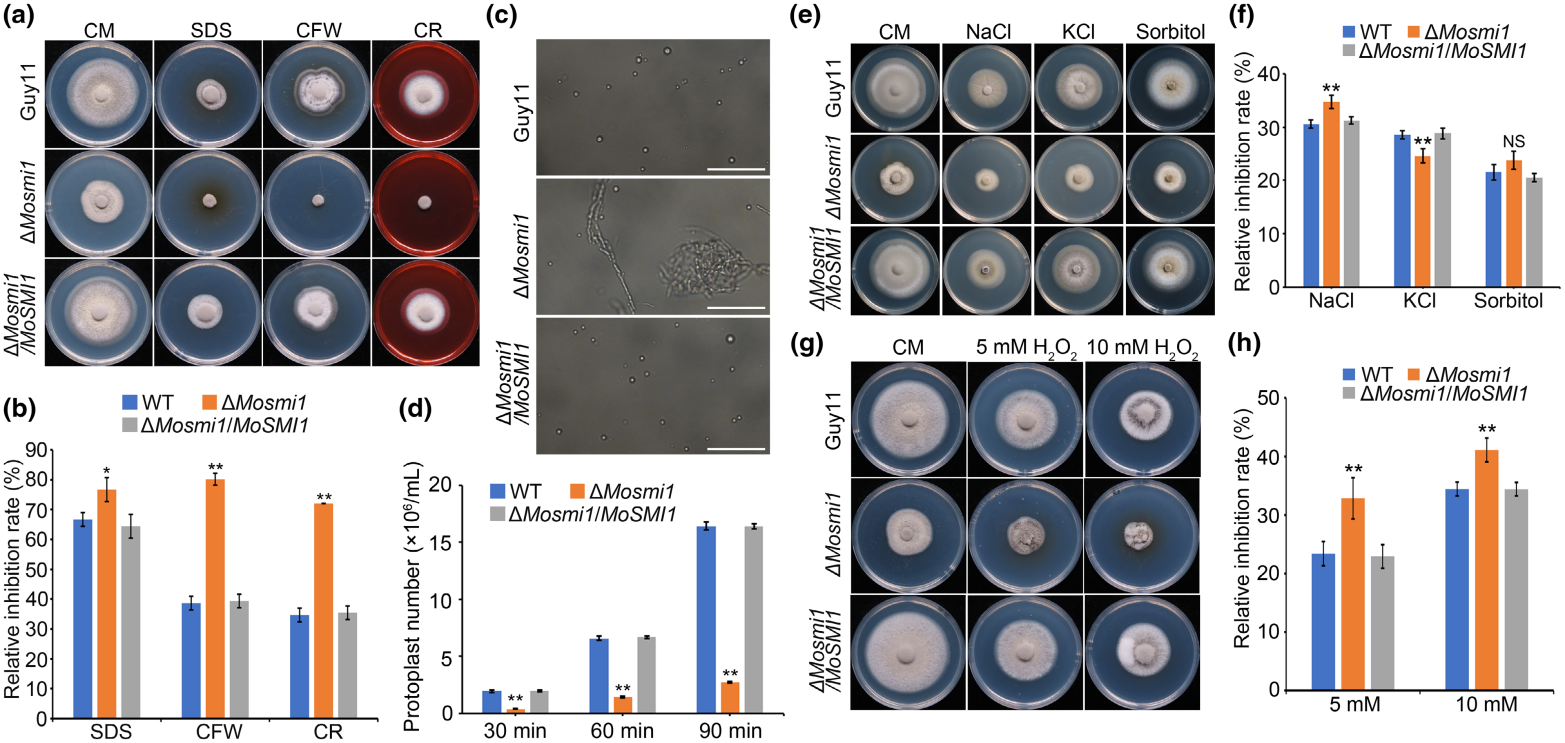
MoSmi1 is required for cell wall integrity and stress response. (a) Colony morphology of all the tested strains on complete medium (CM) plates supplemented with 200 μg/mL calcofluor white (CFW), 600 μg/mL Congo red (CR) or 0.004% sodium dodecyl sulphate (SDS). (b) Statistical analysis of the relative inhibition rate (%) of the tested strains. (c) Protoplasts of all tested strains were observed and photographed after treatment with cell wall‐degrading enzymes for 60 min at 30°C. Bar, 25 μm. (d) Statistical analysis of the protoplast number. Protoplast numbers was calculated at 30, 60 and 90 min. (e) Colony morphology of all the tested strains on CM plates supplemented with 1 M sorbitol, 0.7 M NaCl, or 0.6 M KCl. The colonies were measured and photographed at 7 days post‐inoculation (dpi). (f) Statistical analysis of the relative inhibition rate (%) of the tested strains. (g) Colony morphology of all the tested strains on CM plates supplemented with 5 mM H_2_O_2_ or 10 mM H_2_O_2_. The colonies were measured and photographed at 7 dpi. (h) Statistical analysis of the relative inhibition rate (%) of the tested strains. For each strain, three independent biological experiments were performed with four replicates. Error bars represent *SD*, and asterisks above the columns indicate significant differences between the wild‐type (WT) Guy11 and Δ*Mosmi1* mutant estimated by Student's *t* test (*p < 0.05, **p < 0.01) and NS indicates non‐significant differences between the wild‐type Guy11 and Δ*Mosmi1* mutant.

To investigate the contribution of *MoSMI1* to the CWI of *M. oryzae*, we observed the mycelial growth of all tested strains on CM supplemented with cell wall stress agents (600 μg/mL Congo red [CR], 200 μg/mL CFW, and 0.004% sodium dodecyl sulphate [SDS]). At 7 dpi, the colony diameters of all tested strains were measured. The results showed that the relative inhibition rate of the Δ*Mosmi1* mutant was significantly higher than that of the wild‐type strain Guy11 and the complemented strainΔ*Mosmi1/MoSMI1* under cell wall stress conditions (Figure 6a,b). In addition, we performed protoplast release assays with cell wall‐lysing enzyme to examine whether *MoSMI1* plays a crucial role in the maintenance of CWI. When the mycelia of all tested strains were treated with cell wall‐lysing enzyme, fewer protoplasts were generated in the Δ*Mosmi1* mutant than in the wild‐type strain Guy11 and the complemented strain Δ*Mosmi1/MoSMI1* after incubation for 30, 60, and 90 min (Figure 6c,d). Taken together, these results indicate that MoSmi1 is involved in maintaining CWI in *M. oryzae*.

To investigate the contribution of *MoSMI1* in environmental stress tolerance in *M. oryzae*, we observed the mycelia growth of all tested strains on CM supplemented with osmotic stress agents (0.7 M NaCl, 1 M sorbitol, and 0.6 M KCl) and oxidative stress agents (5 mM and 10 mM H_2_O_2_). At 7 dpi, the colony diameters of all tested strains were measured. We found that the Δ*Mosmi1* mutant was more sensitivity to 0.7 M NaCl, whereas it was more resistant to 0.6 M KCl, but not 1 M sorbitol (Figure 6e,f), and sensitive to both 5 mM and 10 mM H_2_O_2_ concentrations (Figure 6g,h). Our results indicate that *MoSMI1* plays an important role in stress response.

### 2.9 MoSmi1 interacts with MoOsm1 and MoMps1, and regulates their phosphorylation level in *M. oryzae*

**FIGURE 7:**
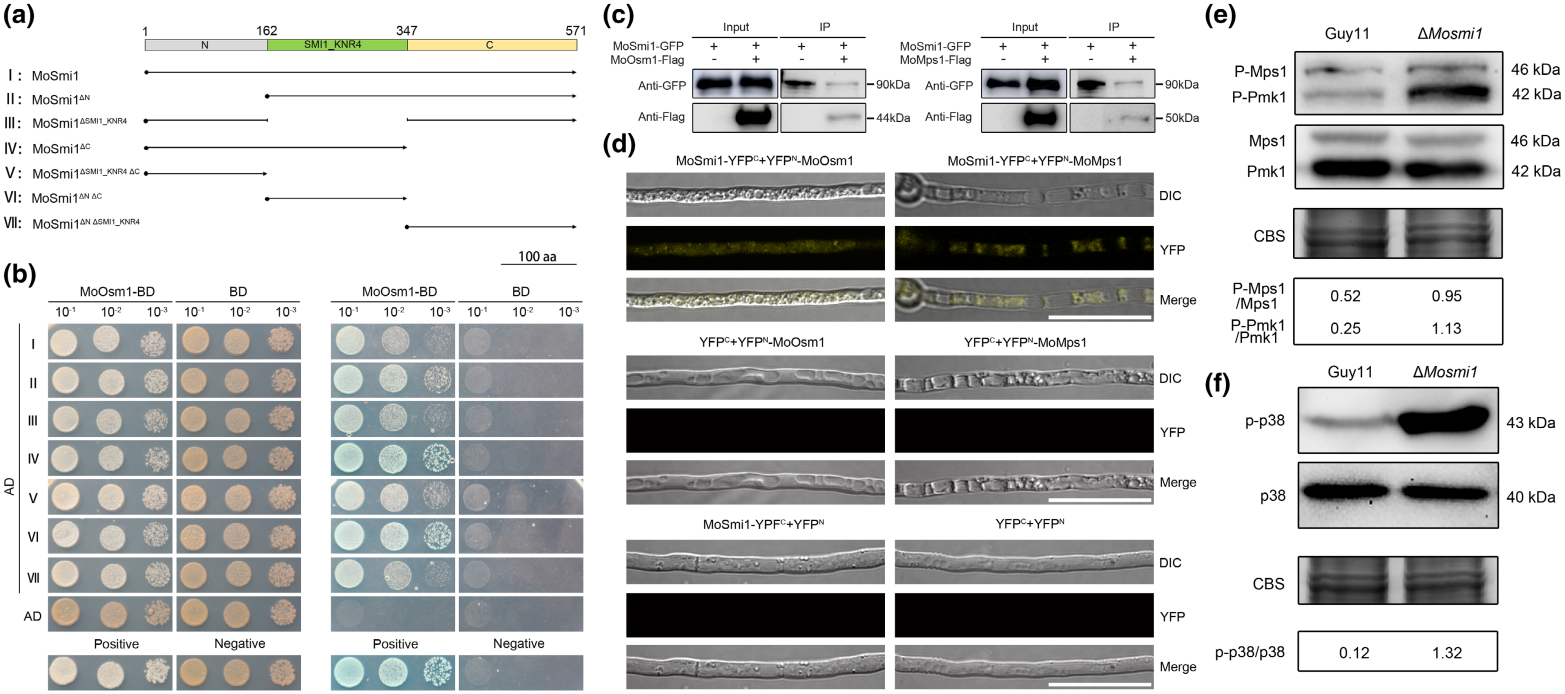
MoSmi1 interacts with MoOsm1 and MoMps1, and regulates their phosphorylation in *Magnaporthe oryzae*. (a) Domain map of MoSmi1. I, full‐length of MoSmi1; II, deletion N‐terminal domain; III, deletion SMI1_KNR4 domain; IV, deletion C‐terminal domain; V, deletion SMI1_KNR4 and C‐terminal domains; VI, deletion of N‐terminal and C‐terminal domains; VII, deletion of N‐terminal and SMI1_KNR4 domains. The domain prediction of MoSmi1 was performed with the SMART analysis. (b) Yeast two‐hybrid assay of seven MoSmi1 variants and MoOsm1. Pairs of different combinations of the truncated constructs of MoSmi1 and MoOsm1 were co‐transformed into yeast strain Y2H Gold and cultured in SD−Leu−Trp and SD−Ade−His−Leu−Trp medium added with X‐α‐gal. (c) Co‐immunoprecipitation assay for the interactions between MoSmi1 and MoOsm1/MoMps1. MoSmi1‐GFP and MoOsm1‐3 × FLAG/MoMps1‐3 × FLAG were expressed in the wild‐type strain Guy11. The experiment was performed with anti‐GFP beads, and the eluted protein was analysed by western blotting using anti‐FLAG and anti‐GFP antibodies. (d) Bimolecular fluorescence complementation assay for interactions between MoSmi1 and MoOsm1/MoMps1. YFP signals was detected in vegetative hyphae expressing MoSmi1‐YFP^C^ and YFP^N^‐MoOsm1/YFP^N^‐MoMps1. The strains expressing YFP^C^ and YFP^N^‐MoOsm1, YFP^C^ and YFP^N^‐MoMps1, MoSmi1‐YFP^C^ and YFP^N^, or YFP^C^ and YFP^N^ were used as negative controls. Bar, 10 μm. (e) Analysis of Pmk1 and Mps1 phosphorylation levels. Phosphorylated Pmk1 and Mps1 were detected using antibodies anti‐Phospho‐p44/42 MAPK and anti‐Phospho‐p42 antibody. (f) Analysis of the phosphorylation level of Osm1. Phosphorylated Osm1 was detected using p‐p38 MAPK antibody and p38 antibody. CBS indicates Coomassie brilliant‐blue staining.

To explore the underlying mechanism of the function of *MoSMI1*, we performed immunoprecipitation combined with mass spectrometry analysis (IP‐MS) to identify the proteins that interact with MoSmi1 (Table S1). To confirm the interaction between MoSmi1 and MoOsm1 or MoMps1, the yeast two‐hybrid (Y2H) assay was performed, which demonstrated that MoSmi1 interacts with MoOsm1 but not with MoMps1(Figure 7a,b, Figure S6). We inferred that Y2H may not be sufficiently sensitive to detect the interaction between MoSmi1 and MoMps1. The different domains of Knr4 have been demonstrated to play diverse roles in protein–protein interactions (Dagkessamanskaia, Durand, et al., 2010). To explore whether MoSmi1 domains have different effects on the interaction with MoOsm1, we generated six MoSmi1 derivative constructs and paired them with MoOsm1, which were then co‐transformed into the yeast strain Y2H Gold. These results showed that all MoSmi1 derivatives interacted with MoOsm1. We also observed that the MoSmi1–MoOsm1 interaction was greatly enhanced when the C‐terminal domain of MoSmi1 was absent, indicating that the C‐terminal domain could partially suppress the MoSmi1–MoOsm1 interaction (Figure 7a,b). Additionally, the interaction between MoSmi1 with MoOsm1 and MoMps1 was detected using co‐immunoprecipitation (Co‐IP) and bimolecular fluorescence complementation (BiFC). The results indicated that MoSmi1 interacted with MoOsm1 and MoMps1 (Figure 7c,d).

Given that MoSmi1 interacts with the protein kinase MoOsm1 and MoMps1, we wondered whether the activity of MoSmi1 may be regulated by MoOsm1 or MoMps1 through protein phosphorylation. Therefore, we determined the phosphorylation level of MoSmi1 by Mn^2+^‐Phos‐tag SDS‐PAGE. The putative *MoMPS1* deletion mutants were verified by PCR and RT‐qPCR, and were named the Δ*Momps1* mutant (Figure S7). The *MoSMI1‐GFP* constructs were transferred into the wild‐type strain Guy11, Δ*Moosm1* and Δ*Momps1* mutant. The MoSmi1‐GFP protein was extracted from the wild‐type strain Guy11, Δ*Moosm1* and Δ*Momps1* mutants, and treated with phosphatase or phosphatase inhibitor. Protein samples were separated by Mn^2+^‐Phos‐tag SDS‐PAGE and detected by immunoblotting with an anti‐GFP antibody. The mobility of MoSmi1‐GFP was similar in all samples (Figure S8), indicating that MoOsm1 or MoMps1 could not regulate MoSmi1 through protein phosphorylation.

In *M. oryzae*, MAPK signalling pathways regulate development, appressorium formation, appressorium‐mediated penetration, stress resistance, and pathogenicity (Cai et al., 2022; Tucker & Talbot, 2001). Among these MAPK signalling pathways, the Pmk1 MAP kinase pathway is important for appressorium formation and plant infection (Xu & Hamer, 1996; Zhao et al., 2007). The Mps1 MAP kinase pathway regulates CWI, penetration, and infection (Li et al., 2012). The Osm1 MAP kinase pathway is mainly responsible for the osmotic stress response (Li et al., 2012). As shown by the above results, the Δ*Mosmi1* mutant exhibited defects in appressoria formation, CWI, osmotic response, and penetration. Therefore, we performed western blotting to determine the phosphorylation levels of Pmk1, Mps1 and Osm1. Compared with the wild‐type Guy11, the phosphorylation levels of Pmk1, Mps1 and Osm1 were higher in the Δ*Mosmi1* mutant (Figure 7e,f), suggesting that MoSmi1 mediates appressoria formation, CWI, osmotic response, and penetration by regulating the phosphorylation levels of Pmk1, Mps1 and Osm1. Taken together, these results indicate that MoSmi1 interacts with the protein kinase MoOsm1 and MoMps1, and regulates their phosphorylation levels in *M. oryzae*.

### 2.10 *MoSMI1* regulates various metabolic pathways in *M. oryzae*

**FIGURE 8:**
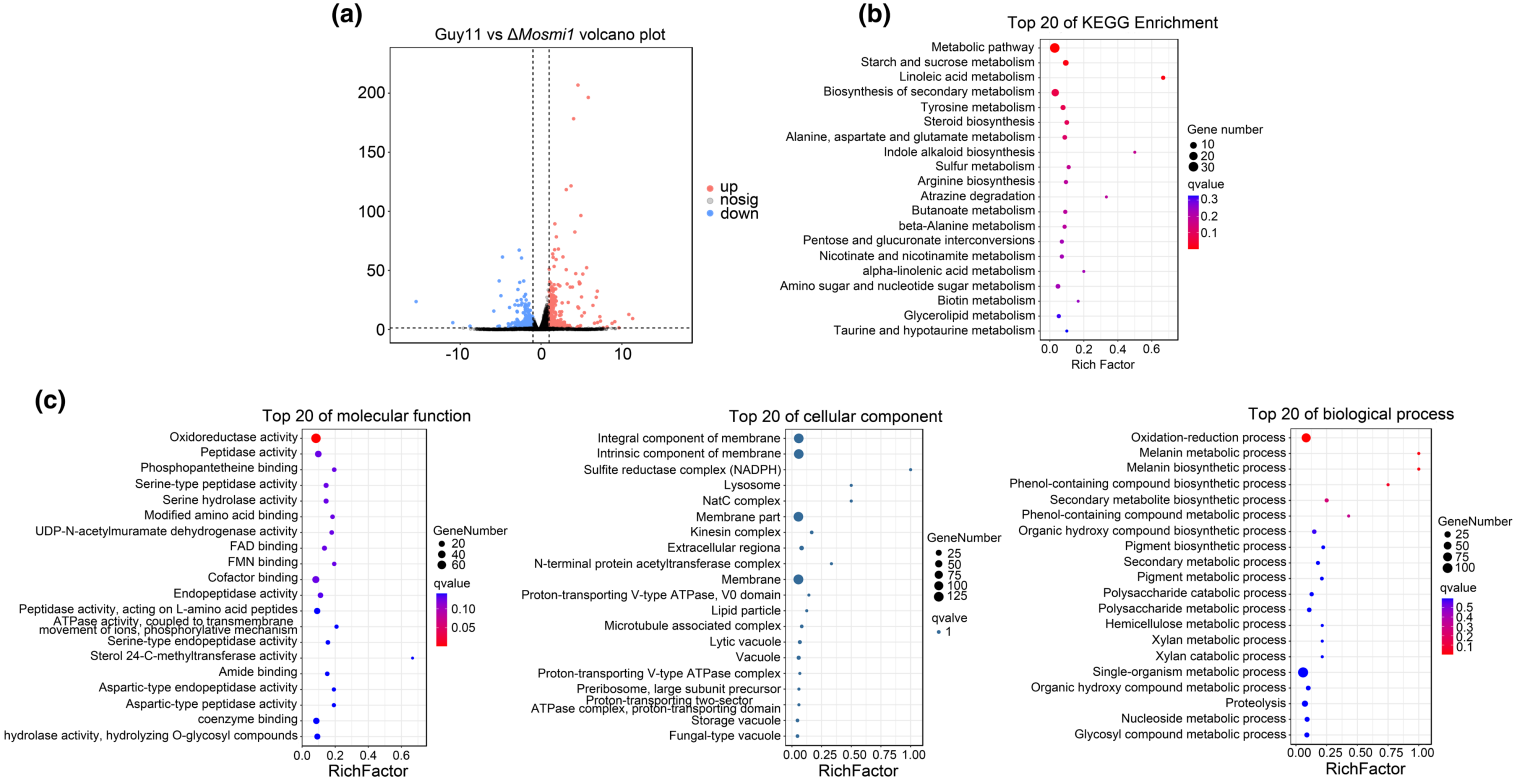
(a) Global view of expressed genes in the wild‐type strain Guy11 and Δ*Mosmi1* mutant strain. (b) Comparison of upregulated and downregulated differentially expressed genes (DEGs) between the wild‐type strain Guy11 and Δ*Mosmi1* mutant strains. (c) Top 20 pathways from KEGG pathway and GO pathway enrichment analysis of significantly upregulated and downregulated genes.

To further investigate the potential regulatory mechanism of *MoSMI1*, we performed transcriptome analysis of the wild‐type strain Guy11 and Δ*Mosmi1* mutant mycelia using RNA‐seq. A total of 610 differentially expressed genes (DEGs) (false discovery rate [FDR] <0.05 and log_2_(fold change [FC]) >1) were identified in the Δ*Mosmi1* mutant compared to the wild type Guy11, 278 of which were upregulated genes and 332 downregulated (Figure 8a, Table S2). The MoSmi1‐encoding gene *MGG_03970* was significantly downregulated, suggesting the RNA‐seq data were reliable (FDR = 2.3e−24, log_2_FC = −15; Table S2). Kyoto Encyclopedia of Genes and Genomes (KEGG) and Gene Ontology (GO) enrichment analyses indicated that various pathways or biological processes, such as metabolic pathway, membrane‐related pathway and oxidation–reduction process were regulated by *MoSMI1* (Figure 8b,c). To confirm the authenticity of the RNA‐seq results, 15 DEGs were randomly selected for RT‐qPCR analysis, and the results were consistent with those of the transcriptome analysis (Table S3).

## 3 DISCUSSION

Adaptation to environmental stress is importance for the survival and colonization of pathogen. The cell wall is the first barrier between the cell and the external environment. Disordered CWI contributes to multiple fungal phenotypic defects. Knr4 has been identified as a hub protein conserved among fungi and response to cell wall stresses. In *Candida albicans*, the Δ*smi1* mutant shows a clear hypersensitivity to CFW or SDS treatment and affects the cell wall β‐glucan synthesis (Nett et al., 2011). In *S. cerevisiae*, cell wall biosynthesis is important for cell structure and morphology (Cabib et al., 2001; Orlean, 2012). In *S. cerevisiae*, *KNR4* is not essential for growth under standard laboratory conditions (30°C, rich medium). However, its deletion leads to growth defects under numerous stresses, such as elevated temperature, SDS, caffeine, and various cell wall disrupting agents (Martin‐Yken et al., 2016). In this study, we found that MoSmi1 regulated the CWI pathway, conidiation and morphogenesis of conidia and hyphae.

Evidence has suggested that microtubules play a role in cell cycle progression and nuclear division (Hughes et al., 2008; Jansen et al., 2023). Nuclear positioning through microtubule dynamics is an essential process for many types of cells (Gundersen & Worman, 2013). In fission yeast cells, microtubules are used to reposition nuclei (Bellingham‐Johnstun et al., 2023). In *Arabidopsis thaliana*, transport protein particle II (TRAPPII) tethering factors interact with the microtubule‐associated proteins of the PLEIADE/AtMAP65 family, which are required to coordinate cytokinesis with the nuclear division cycle (Steiner et al., 2016). Microtubules also play an important role in cell morphology and material transportation. In mammals, the stability of disk‐like septins depends on intact microtubules (Sellin et al., 2011). In filamentous fungi, cytoplasmic microtubules serve as highways for the long‐distance bidirectional transport of organelles, mRNA, and other subcellular cargos (Abenza et al., 2009). In short, microtubules play roles in cell cycle regulation, cell morphology, and material trafficking. Here, we determined that MoSmi1 plays a vital role in microtubule organization and cytoplasmic division, which are key factors of cell morphogenesis in *M. oryzae*. Moreover, the deletion of *MoSMI1* resulted in a significantly decreased appressorium formation rate, which led to a lower penetration rate and weakened pathogenicity. We also found that MoSmi1 participates in the functional septin ring organization during the appressoria maturation.

Cell morphogenesis in eukaryotes is a complex process that requires perfect coordination of several different regulation and signal transduction pathways. MAPK is a family of protein kinases that regulate proliferation, gene expression, differentiation, mitosis, survival, and apoptosis in a wide variety of organisms (Avruch, 2007; Manning et al., 2002). In *S. cerevisiae*, Knr4 was identified as a member of the PKC1‐mediated MAPK cascade signalling pathway and is involved in the regulation of cell cycle progression by cooperation with Bck2 (Martin‐Yken et al., 2002). In addition, Knr4 interacts with the Slt2 MAP kinase, a key component of the CWI pathway, which has been shown to affect the transcriptional outputs of CWI pathway (Martin‐Yken et al., 2003, 2016). Moreover, the MAP kinase Hog1 is activated in *smi1*Δ cells and the activation of Hog1 induces the translocation of Msn2 into the nucleus. The nuclear accumulation of Msn2 enhances Sir2‐mediated rDNA stability and affects yeast cell cycle progression (Hong & Huh, 2021). In *M. oryzae*, osmotic stress regulates the nuclear localization pattern of MoHog1 and MoMsn2, activating the transcription of the target genes in response to environmental stresses (Bohnert et al., 2019; Zhang et al., 2014). In this study, we found that MoSmi1 interacts with MoMps1 and MoOsm1, which are core components of the Mps1 MAPK and Osm1 MAPK signalling pathways in *M. oryzae*. In addition, the phosphorylation levels of MoMps1, MoOsm1, and MoPmk1 were significantly increased in the Δ*Mosmi1* mutant, suggesting that MoSmi1 regulates growth, development, appressorium maturation, and virulence by mediating the MAPK signalling pathway.

In *S. cerevisiae*, a role of Knr4 in gene expression was first revealed by its ability to simultaneously repress all three chitin synthase genes upon overexpression (Martin et al., 1999). Smi1 was also found to regulate the transcription of genes involved in the cell cycle, cell wall synthesis, morphogenesis, and transcriptional responses to heat and cell wall stress in different fungi (Lagorce et al., 2003; Martin‐Yken et al., 2002; Penacho et al., 2012). Smi1, as a regulator of glucan synthases and glucanases, mediates the trafficking, stability, and localization of Bgs4, which is important for growth, response to stress, and cytoplasmic division (Longo et al., 2022). In our study, transcriptome data suggested that MoSmi1 is involved in regulation of membrane‐related pathways and oxidation–reduction process, which is associated with the response to oxidative stress and the partial localization of the membrane of MoSmi1.

Hub proteins are intrinsically disordered proteins (IDPs) or contain IDRs, which allow them to be involved in multiple interactions and various biological functions (Dunker et al., 2005; Uversky, 2013). Knr4 contains a large IDR and is considered to be an important hub of the yeast interactome (Martin‐Yken et al., 2016). The N‐terminal domain of Knr4 is required for the interaction of Knr4 with several partners (Dagkessamanskaia, Durand, et al., 2010; Fino et al., 2010). Additionally, deletion of the C‐terminal domain greatly enhances the Knr4‐Slt2 interaction, and the N‐terminal region is required for the interaction to occur (Batista et al., 2023). In this study, we predicted three IDRs, two of them positioned in the C‐terminal domain and the other in the SMI1_KNR4 domain. In addition, we verified that the deletion of any one domain did not prevent the Smi1–Osm1 interaction and that the loss of the C‐terminal domain could promote the Smi1–Osm1 interaction. Furthermore, IDRs has been shown to mediate protein phase separation in several studies (Lindstrom et al., 2022; Majumdar et al., 2019; Saito et al., 2019; Schuster et al., 2020; Wang et al., 2018). Phase separation can prompt non‐membrane‐bound components to form compartments, such as stress granules (SGs), P‐body (PB), and nucleolus, to separate from the surrounding environment, and the components within it can diffuse freely so that chemical reactions can take place inside (Hyman et al., 2014). In *S. cerevisiae*, liquid–liquid phase separation was identified to promote formation of foci under stress, which provides valuable clues for understanding the mechanisms underlying SG formation and SG‐associated human diseases (Lindstrom et al., 2022). The IDRs of MoSmi1 may be related to its ability to respond to stress, which may be mediated by phase separation. In future, we may provide novel strategies to control rice blast by connecting phase separation with the pathogenic mechanism of *M. oryzae*.

Taken together, our study revealed that MoSmi1 regulates CWI by modulating MAPK signalling pathways to affect cell morphology, cytoskeletal dynamics, cell cycle progression, stress response, and pathogenicity of *M. oryzae*.

## 4 EXPERIMENTAL PROCEDURES

### 4.1 Strains and culture conditions

We used Guy11 as the wild‐type (WT) strain in this study. All strains were cultured on complete medium (CM) (10 g d‐glucose, 2 g peptone, 1 g yeast extract, 1 g casamino acids, 50 mL 20× nitrate salts, 1 mL vitamin, 1 mL trace elements, 15 g agar, and water added to 1 L) at 28°C under dark conditions. For DNA, RNA, and protein extraction, all tested strains were grown in liquid CM in a rotatory shaker at 120 rpm, and 28°C for 48 h.

### 4.2 Plasmid construction and genetic transformation

To generate the target gene deletion mutants, the 1.2 kb upstream fragment and downstream fragment of the target gene were amplified from the genomic DNA of the wild‐type strain Guy11 using primers (Table S4). The hygromycin resistance gene (*HPH*) fragment was amplified from pFGL821 using primers HF/HR. All fragments were cloned into the HindIII/XbaI‐linearized vector pKO1B using a one‐step cloning kit (Vazyme) to obtain a gene‐knockout vector. The knockout vector was transformed into the wild‐type strain Guy11 using *Agrobacterium tumefaciens*‐mediated transformation (ATMT). Transformants were screened using CM plates containing 250 μg/mL hygromycin B and further verified by PCR and RT‐PCR using the pairs listed in Table S4. Positive transformants were further confirmed by Southern blotting or RT‐qPCR. Two independent mutants with similar phenotypes were obtained and one was selected for further experiments.

For the complemented strain Δ*Mosmi1/MoSMI1*, an approximately 1.5 kb sequence containing the native promoter and open reading frame (ORF) of *MoSMI1* without a stop codon was amplified from the genomic DNA of the wild‐type strain Guy11 using primer pairs listed in Table S4. The PCR products were cloned into yeast strain XK1‐25 with XhoI‐linearized vector pYF11 to obtain a complemented vector pYF11‐MoSmi1. The construct was transformed into Δ*Mosmi1* mutant strain using polyethylene glycol (PEG)‐mediated transformation. Transformants were screened using CM plates containing 60 μg/mL bleomycin and further verified by fluorescence. The positive transformants were further verified by RT‐PCR using primer pairs listed in Table S4. The phenotypes of all transformants recovered to those of the wild‐type strain Guy11, and one of them was selected for further experiments. The same method was used to obtain the wild‐type strain Guy11 and Δ*Mosmi1* expressing β‐tubulin‐pYF11, Sep3‐pYF11, Sep5‐pYF11, respectively. To observe the localization of β‐tubulin, an approximately 1.5 kb sequence containing the native promoter and open reading frame (ORF) of the *β‐tubulin* gene without a stop codon was amplified from the genomic DNA of the wild‐type strain Guy11 using primer pairs listed in Table S4. PCR products were cloned into KpnI/XhoI‐linearized pFGL820 (fused with RFP). The construct was transformed into the wild‐type strain Guy11, Δ*Mosmi1* mutant strain, and complemented strain Δ*Mosmi1/MoSMI1* using ATMT method. Transformants were screened on medium (1.7 g yeast nitrogen base without amino acids, 10 g d‐glucose, 2 g asparagine, 1 g NH_4_NO_3_, 15 g agar, and water added to 1 L) containing 40 μg/mL chlorimuron‐ethyl and further verified by fluorescence.

### 4.3 RT‐qPCR analysis

Total RNA of the tested strains was isolated from fresh mycelial pellets using RNeasy Mini Kit (Qiagen). For cDNA synthesis, 5 μg total RNA from each strain was reverse‐transcribed using HiScript II 1st Strand cDNA Synthesis Kit (+gDNA wiper) (Vazyme). RT‐PCR was performed to confirm the deletion and complementation of the targeted gene using the gene‐specific primers listed in Table S4. The *ACTIN* gene (*MGG_03982*) was used as an endogenous reference. qPCR was performed with ChamQ Universal SYBR qPCR Master Mix (Vazyme) using CFX Connect Real‐Time PCR Detection System (Bio‐Rad). The β‐tubulin gene (*MGG_00604*) was used as an endogenous reference. The experiment was repeated three times with three replicates each time. All primers used in the assays are listed in Table S4.

### 4.4 Assays for vegetative growth, conidiation, appressorium formation, and stress agents

For growth assay, mycelial blocks of the wild‐type strain Guy11, Δ*Mosmi1* mutant, and complemented strain Δ*Mosmi1/MoSMI1* were cultured on CM plates at 28°C under dark conditions. After 7 days, the colony diameter was measured, and all tested strains were photographed using a camera (Nikon). For conidiation analysis, the wild‐type strain Guy11, Δ*Mosmi1* mutant, and complemented strain Δ*Mosmi1/MoSMI1* were cultured on rice decoction and cornmeal (RDC) medium at 28°C for 5 days in the dark, followed by 3 days of continuous illumination under fluorescent light. Subsequently, 10 mL water was used to collect conidia, and the conidial suspensions were condensed to 1 mL. The number of conidia was counted with a haemocytometer under a microscope. For appressorium formation analysis, conidial suspensions of all tested strains were induced on artificial hydrophobic surfaces at 28°C in the dark. The appressorium formation rate was detected after 6, 12, and 24 h. Images of appressorium formation were obtained using an inverted fluorescence microscope (Nikon).

To determine strains' response to various stress agents, the wild‐type strain Guy11, Δ*Mosmi1* mutant, and the complemented strain Δ*Mosmi1/MoSMI1* were cultured on CM plates supplemented with 15 μg/mL benomyl, 1 M sorbitol, 0.7 M NaCl, 0.6 M KCl, 5 mM H_2_O_2_, 10 mM H_2_O_2_, 200 μg/mL CFW, 600 μg/mL CR, or 0.004% SDS at 28°C in dark. Subsequently, the colonies were photographed and the diameter of the colonies was measured after 7 days. Relative inhibition rate = (diameter of the untreated strain − diameter of the strain treated with chemicals)/(diameter of the untreated strain). All experiments were repeated three times with three replicates each time.

### 4.5 Staining assay

To observe the vacuole localization of MoSmi1‐GFP during appressorium formation, the appressoria from the wild type expressing MoSmi1‐GFP were stained with 10 mM CMAC (Invitrogen) at room temperature for 30 min under dark conditions and observed under a laser scanning confocal microscope. To observe the morphology of conidia, conidia (5 ×  10^4^ conidia/mL) of all tested strains were stained with 10 μg/mL CFW (Sigma‐Aldrich) solution for 10 min and photographed under a laser scanning confocal microscope (Nikon). For the ROS staining assay, barley leaves infected with a conidial suspension of all tested strains at 30 hpi were stained with 1 mg/mL DAB (Sigma‐Aldrich) solution (pH 3.8) for 12 h, and then destained with ethanol for 4 h on a shaker at 28°C. For ROS inhibition assay, 0.5 μM DPI (Sigma‐Aldrich) (dissolved in DMSO) was added to a conidial suspension of all tested strains to inhibit host ROS. DMSO was used as control. Barley epidermal cells were observed under an inverted fluorescence microscope (Nikon). To determine the cytoplasmic division of the wild‐type strain Guy11 and the Δ*Mosmi1* mutant, the mycelia of strains expressing H1‐RFP were stained with 10 μg/mL CFW (Sigma‐Aldrich) solution for 10 min and photographed under a laser scanning confocal microscope (Nikon).

### 4.6 Virulence assays

For the virulence study, the rice cultivar CO39 and the barley cultivar Golden Promise were used. The mycelial blocks or conidial suspensions (5 × 10^4^ conidia/mL) of all tested strains were inoculated on 7‐day‐old isolated barley leaves and kept in a biological incubator at 28°C with 90% humidity in the dark for the first 24 h, followed by a 12/12 h light/dark cycle. The lesion spots were observed and captured at 5 dpi. The lesion areas of all tested strains were analysed using ImageJ software. Approximately 15 mL conidial suspensions (5 × 10^4^ conidia/mL in a 0.2% wt/vol gelatin solution) from each tested strain were sprayed onto 14‐day‐old rice seedlings and kept in a biological incubator at 28° with 90% humidity in the dark for the first 24 h, followed by a 12/12 h light/dark cycle. The disease spots were observed and photographed at 5 dpi. Three leaves from each strain were used for statistical analysis of the number of lesions.

To observe the expansion of invasive hyphae (IH), conidial suspensions (5 × 10^4^ conidia/mL) of the tested strains were dropped on the back of barley leaves and kept in a biological incubator at 28°C with 90% humidity under the dark. Barley epidermal cells were observed at 24, 36, and 48 hpi using an inverted fluorescence microscope (Nikon). All the above experiments were repeated three times with three replicates each time.

### 4.7 Protoplast release assay

All the tested strains were cultured in liquid CM at 28°C for 48 h. Then, 0.2 g of mycelia were collected and treated with 0.01 g/mL cellulase, 0.01 g/mL pectinase, and 0.0035 g/mL driselase (dissolved in 10 mL 0.7 M NaCl solution) for 30, 60, and 90 min at 30°C. The protoplasts were then observed under an inverted fluorescence microscope (Nikon) and counted using a haemocytometer. This experiment was repeated three times with three replicates each time.

### 4.8 Fluorescent microscopic observation

For the subcellular localization assay, the fluorescence signals of conidia, appressorium formation (6, 12, and 24 hpi), and IH (24 hpi) were observed using a laser scanning confocal microscope (Nikon). To analyse the morphology of the microtubules in the wild‐type strain Guy11, Δ*Mosmi1* mutant, and the complemented strain Δ*Mosmi1/MoSMI1*, the fluorescence signals of mycelia were observed using a laser scanning confocal microscope (Nikon). To detect the morphology of the septin ring in the wild‐type strain Guy11 and Δ*Mosmi1* mutant, the fluorescence signals of appressoria (24 hpi) were observed using a laser scanning confocal microscope (Nikon).

### 4.9 Western blot assay

Total protein was extracted from mycelia inoculated in liquid CM for 48 h, as previously described (Zhang et al., 2017). The protein samples were detected by anti‐GFP antibody, anti‐FLAG antibody (Abmart), Phospho‐p44/42 MAPK antibody, p44/42 MAPK antibody (Cell Signalling Technology), Phospho‐p‐p38 MAPK antibody, or p38 MAPK antibody (Santa Cruz Biotechnology).

### 4.10 Affinity purification and mass spectrometry analysis

The pYF11‐MoSmi1 vector was transformed into the wild‐type strain Guy11 through PEG‐mediated transformation. The bleomycin‐resistant transformants were examined under a laser scanning confocal microscope (Nikon), and confirmed by western blotting with anti‐GFP antibody. The total protein of the positive transformants was extracted and incubated with anti‐GFP nanobody agarose beads (AlpalifeBio) for 4 h at 4°C. The bound proteins were eluted from the GFP‐beads and electrophoresed in 10% SDS‐PAGE until proteins were concentrated into the separation gel. The gel containing the protein sample was stained with Coomassie brilliant blue and sent to APTBIO (Shanghai, China) for mass spectrometry analysis as previously described (Zhang et al., 2017).

### 4.11 Yeast‐two hybrid assays

The cDNA fragments of full‐length *MoSMI1* and its domain derivatives, including the N‐terminal deletion region (deletion 1–161 amino acid), SMI1_KNR4 deletion region (deletion 162–347 amino acid), C‐terminal deletion region (deletion 348–571amino acid), N‐terminal domain (remaining 1–161 amino acid), SMI1_KNR4 domain (remaining 162–347 amino acid), and C‐terminal domain (remaining 348–571 amino acid) were amplified using cDNA template of the wild‐type strain Guy11 and cloned into pGADT7 as the prey constructs. The cDNA fragments of *MoOSM1* (*MGG_01822*) and *MoMPS1* (*MGG_04943*) were amplified using cDNA template of the wild‐type strain Guy11 and cloned into pGBKT7 as the bait constructs. Corresponding primers are listed in Table S4. Both the prey construct and bait constructs were co‐transformed into the yeast strain Y2HGold, according to the manufacturer's instructions (Matchmaker Gold Yeast Two‐Hybrid System). The transformants were grown on a synthetic‐defined medium lacking leucine and tryptophan (SD−Leu−Trp) and then transferred to synthetic defined medium lacking adenine, histidine, leucine, and tryptophan (SD−Ade−His−Leu−Trp). The positive control was the interaction between pGBKT7‐53 and pGADT7‐T, and the negative control was the interaction between pGBKT7‐Lam and pGADT7‐T.

### 4.12 Bimolecular fluorescence complementation assay

For the BiFC assay, the *MoSMI1* gene fragment without a stop codon was cloned into BamHI‐linearized pKD2‐YFP^CTF^ (hygromycin B resistance) and *MoOSM1* or *MoMPS1* gene fragments without a stop codon were cloned into XbaI‐linearized pKD5‐YFP^NTF^ (chlorimuron‐ethyl resistance) using a one‐step cloning kit (Vazyme). Corresponding primers are listed in Table S4. Subsequently, pKD2‐MoSmi1‐YFP^CTF^ was co‐transformed into the wild‐type strain Guy11 with pKD5‐MoOsm1‐YFP^NTF^ and pKD5‐MoMps1‐YFP^NTF^ using the ATMT method. The transformants were screened using medium (1.7 g yeast nitrogen base without amino acids, 10 g d‐glucose, 2 g asparagine, 1 g NH_4_NO_3_, 15 g agar, and water added to 1 L) containing 250 μg/mL hygromycin B and 40 μg/mL chlorimuron‐ethyl and further verified by fluorescence. Fluorescence signals were observed using mycelia under a laser scanning confocal microscope (Nikon).

### 4.13 Co‐immunoprecipitation assay

To confirm the interaction between MoSmi1‐MoOsm1 and MoSmi1‐MoMps1 in vivo using Co‐IP, the ORFs of *MoOSM1* or *MoMPS1* without a stop codon were amplified and cloned into HindIII‐linearized pKNRP27Flag (3 × FLAG tag) vector (neomycin resistance) using a one‐step cloning kit (Vazyme). Corresponding primers are listed in Table S4. Subsequently, MoOsm1‐RP27‐3 × FLAG and MoMps1‐RP27‐3 × FLAG were transferred into the wild‐type strain Guy11 expressing pYF11‐MoSmi1 using PEG‐mediated protoplast transformation method. The transformants were screened on CM containing 200 mg/mL G418 and verified by western blotting. The total protein was incubated with GFP agarose beads for 4 h at 4°C. Bound proteins were eluted from GFP‐Trap beads for western blotting analysis. Eluted protein samples were detected using anti‐GFP or anti‐FLAG antibodies (Abmart).

### 4.14 Phosphorylation analysis through Phos‐tag gel electrophoresis

To analyse the phosphorylation level of MoSmi1 in the wild‐type strain Guy11 and Δ*Momps1* or Δ*Moosm1* mutants, pYF11‐MoSmi1 was transformed into the wild‐type strain Guy11, Δ*Moosm1*, and Δ*Momps1* mutants using PEG‐mediated protoplast transformation method. Transformants were screened using CM containing 60 μg/mL bleomycin and further verified by western blotting. The total proteins extracted from mycelia of the tested strains were treated with phosphatase or phosphatase inhibitors. Subsequently, the treated protein samples were electrophoresed in 8% SDS‐PAGE containing 50 mM acrylamide‐dependent Phos‐tag ligand and 100 mM MnCl_2_. Gel electrophoresis was performed at constant voltage (80 V) for 3–4 h. After electrophoresis, the gels were equilibrated twice in transfer buffer with 5 mM EDTA for 20 min, followed by transfer buffer without EDTA for another 20 min. Afterwards, the protein was transferred from gels to a PVDF membrane, which was performed for about 30 h at 80 V at 4°C. The PVDF membrane was analysed by western blotting using an anti‐GFP antibody (Abmart).

### 4.15 Transcriptome analysis

Mycelia of the wild‐type strain Guy11 and Δ*Mosmi1* mutant were collected from liquid CM and sent to GENE DENOVO (Guangdong, China) for RNA extraction and RNA‐seq. Three biological replicates were used for each strain. DEGs were analysed by the DESeq2 package, and expression with log_2_FC > |1| and FDR < 0.05 were defined as DEGs (Love et al., 2014). Ontology enrichment analysis was performed using the topGO R package, and *p* < 0.05 were considered significantly enriched by DEGs (Young et al., 2010). KEGG enrichment analysis was performed using clusterProfiler R package to test the statistical enrichment of DEGs in KEGG pathways (Yu et al., 2012).

### 4.16 Statistical analysis

All results of the statistical analyses are presented as mean ± *SD* of three independent biological replications, along with at least three technical replicates. Statistical significance was determined using a two‐sample Student's *t* test, performed with Microsoft Office Excel software. The *p* value was used to assess the statistical significance of the results (NS, *p* > 0.05, *p < 0.05, **p < 0.01).

## CONFLICT OF INTEREST STATEMENT

The authors have no competing financial interest and solely responsible for the experimental designs and data analysis.

## Supporting information


FigureS1

FigureS2

FigureS3

FigureS4

FigureS5

FigureS6

FigureS7

FigureS8

TableS1

TableS2

TableS3

TableS4

## Data Availability

All data supporting the findings of the current study are available within figures and Supporting Information. All strains generated during this study are available from the corresponding author upon reasonable request.
