# Correction to “A novel MAP kinase‐interacting protein MoSmi1 regulates development and pathogenicity in *Magnaporthe oryzae*”

**DOI:** 10.1111/mpp.13503

**Published:** 2024-08-02

## Abstract

Wang, Y., Cui, X., Xiao, J., Kang, X., Hu, J., Huang, Z. et al. A novel MAP kinase‐interacting protein MoSmi1 regulates development and pathogenicity in *Magnaporthe oryzae*. *Molecular Plant Pathology*. 2024; 25, e13493.

In section 2.1 (Results), first sentence, the reference citation to Zhang, Chen, et al. (2022) should be deleted. The correct sentence is “Through RNA sequencing (RNA‐seq) analysis, we previously found that the gene *MGG_03970* was upregulated in the Δ*Monap1* mutant (Zhang, Wang, et al., 2022).”

In section 4.2 (Experimental Procedures), paragraph 2, seventh sentence, β‐tubulin‐pYF11 should be deleted. The correct sentence is “The same method was used to obtain the wild‐type strain Guy11 expressing Sep3‐pYF11 or Sep5‐pYF11, and Δ*Mosmi1* expressing Sep3‐pYF11 or Sep5‐pYF11.”

We apologize for these errors.

Wang, Y., Cui, X., Xiao, J., Kang, X., Hu, J., Huang, Z. et al. A novel MAP kinase‐interacting protein MoSmi1 regulates development and pathogenicity in *Magnaporthe oryzae*. *Molecular Plant Pathology*. 2024; 25, e13493.

In section 2.1 (Results), first sentence, the reference citation to Zhang, Chen, et al. (2022) should be deleted. The correct sentence is “Through RNA sequencing (RNA‐seq) analysis, we previously found that the gene *MGG_03970* was upregulated in the Δ*Monap1* mutant (Zhang, Wang, et al., 2022).”

In section 4.2 (Experimental Procedures), paragraph 2, seventh sentence, β‐tubulin‐pYF11 should be deleted. The correct sentence is “The same method was used to obtain the wild‐type strain Guy11 expressing Sep3‐pYF11 or Sep5‐pYF11, and Δ*Mosmi1* expressing Sep3‐pYF11 or Sep5‐pYF11.”

We apologize for these errors.

