# Supplementary figures and images for: A novel MAP kinase‐interacting protein MoSmi1 regulates development and pathogenicity in Magnaporthe oryzae
Source: Mol Plant Pathol. 2024 Jul 21;25(7):e13493. doi: 10.1111/mpp.13493 (PMC11260997; doi:10.1111/mpp.13493)

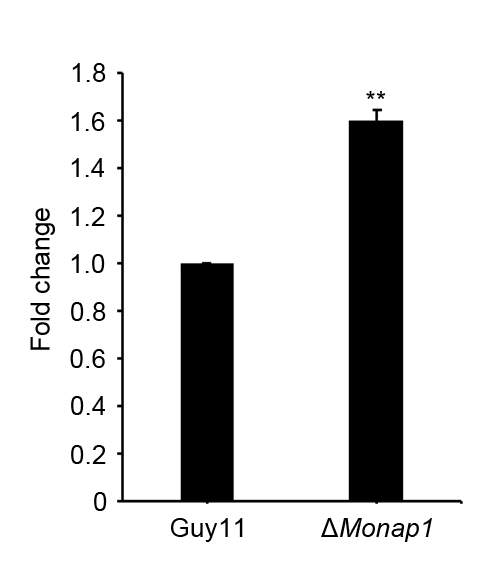

Supplement: Supplementary file 1 — FigureS1 [file MPP-25-e13493-s004.tif]

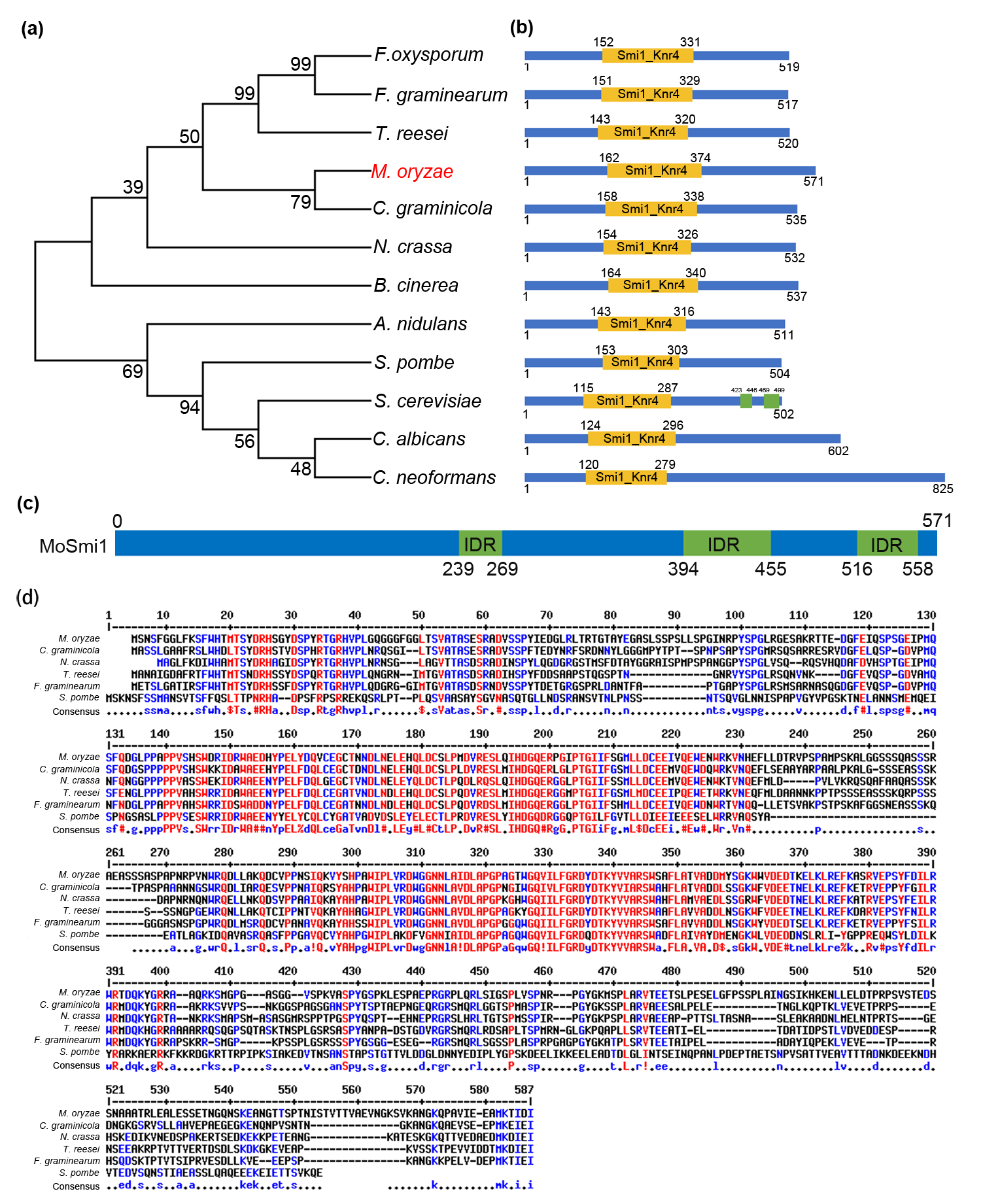

Supplement: Supplementary file 2 — FigureS2 [file MPP-25-e13493-s005.tif]

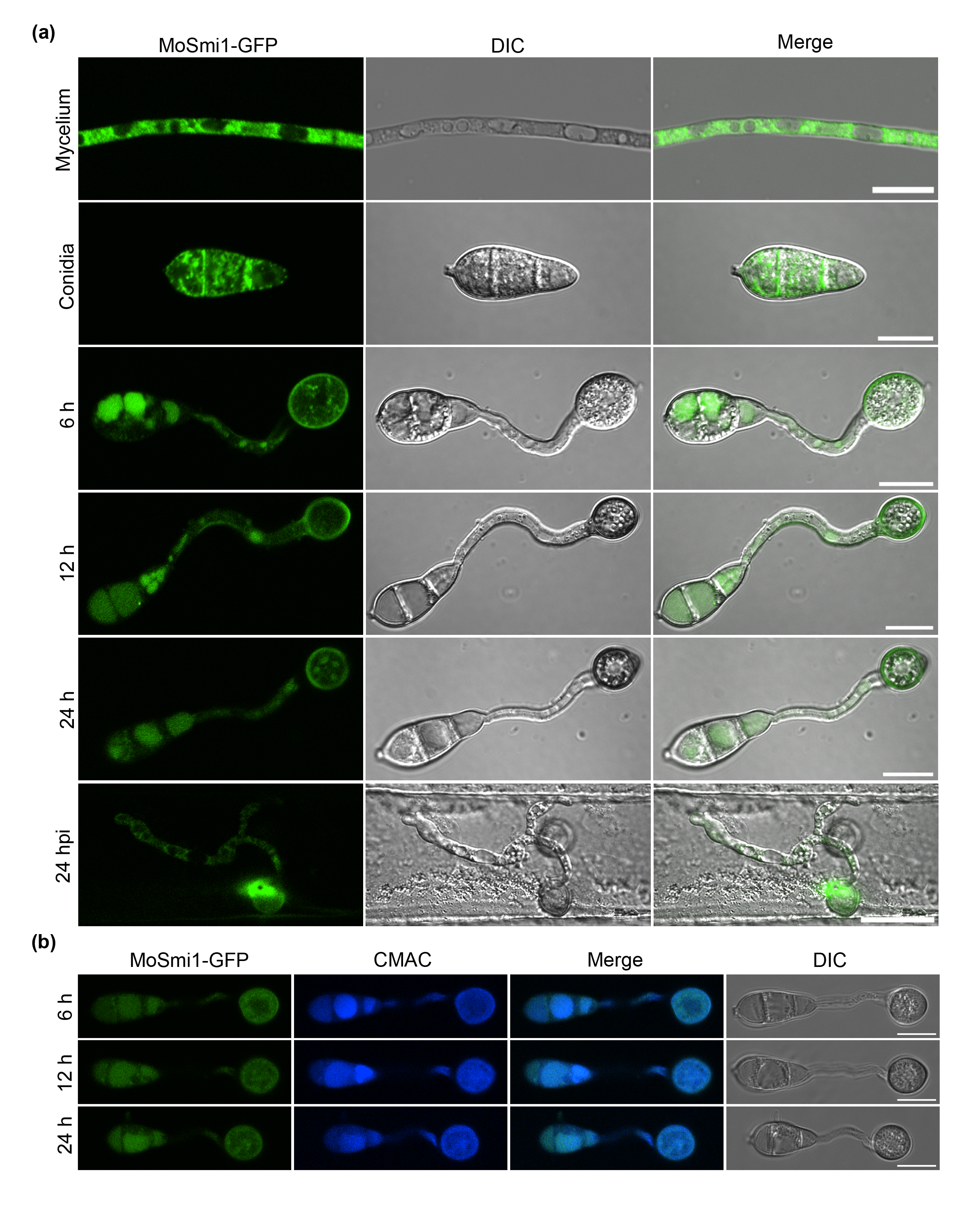

Supplement: Supplementary file 3 — FigureS3 [file MPP-25-e13493-s009.tif]

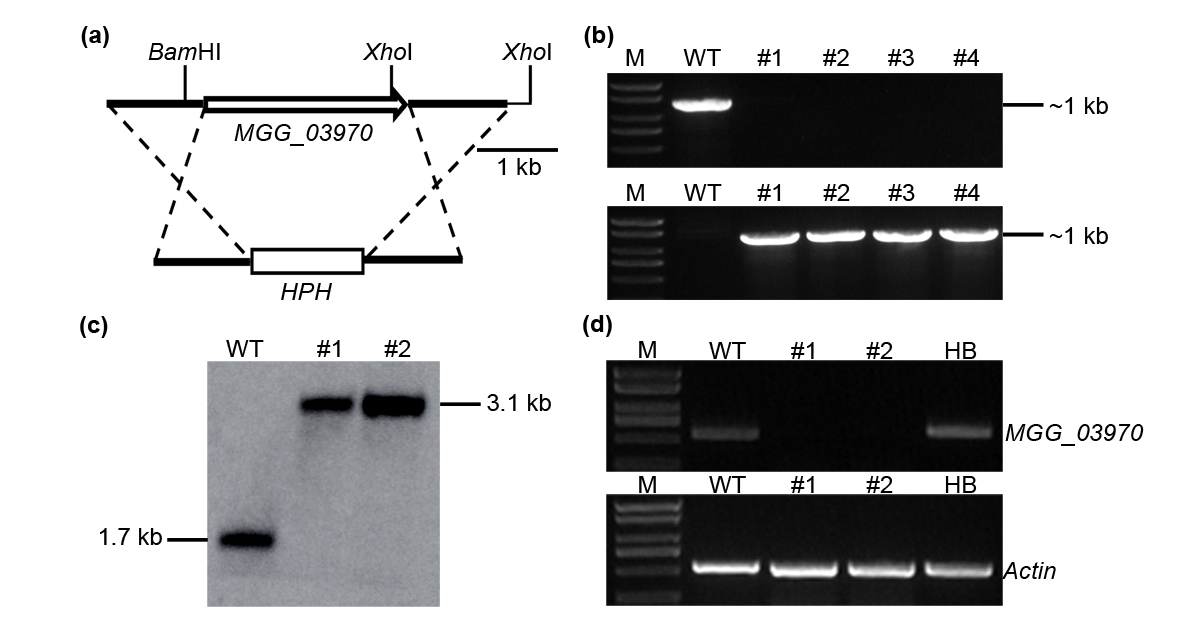

Supplement: Supplementary file 4 — FigureS4 [file MPP-25-e13493-s006.tif]

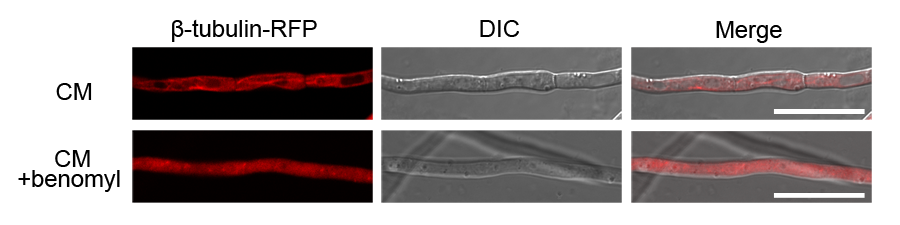

Supplement: Supplementary file 5 — FigureS5 [file MPP-25-e13493-s003.tif]

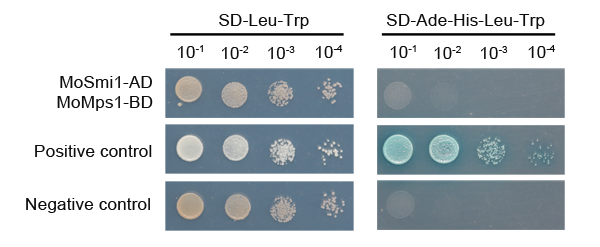

Supplement: Supplementary file 6 — FigureS6 [file MPP-25-e13493-s002.tif]

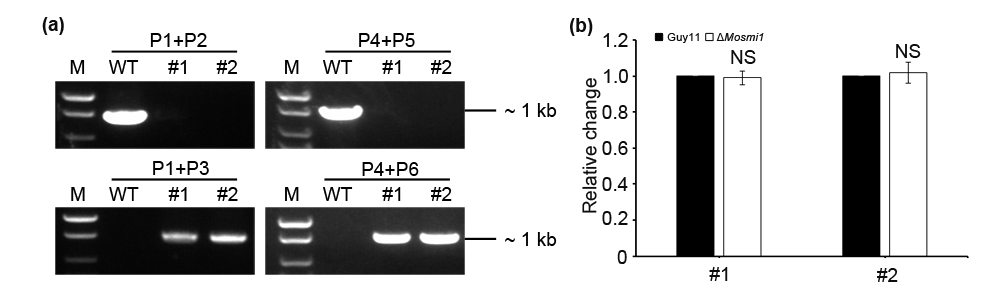

Supplement: Supplementary file 7 — FigureS7 [file MPP-25-e13493-s007.tif]

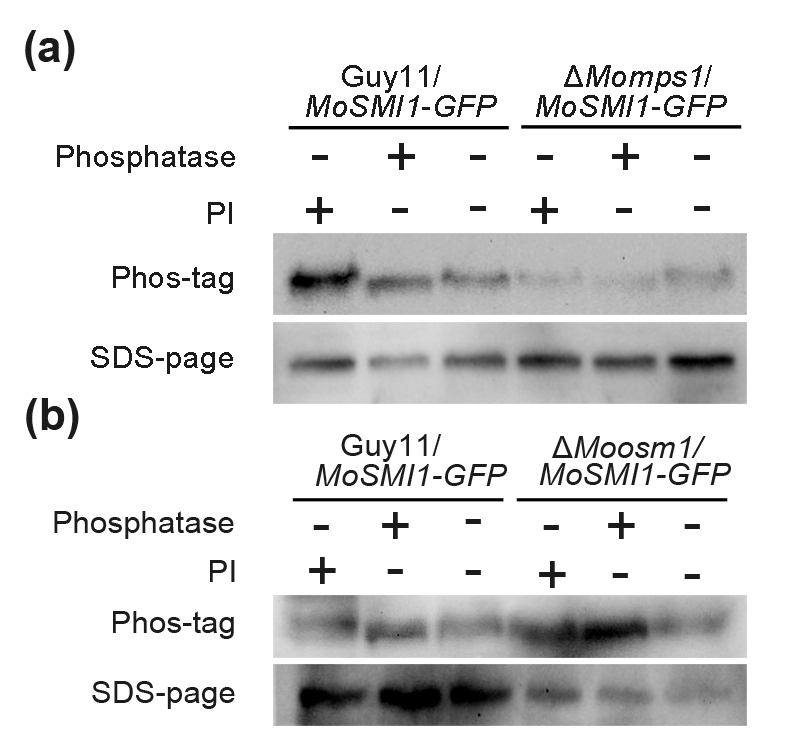

Supplement: Supplementary file 8 — FigureS8 [file MPP-25-e13493-s010.tif]
