# Supplementary material for: A novel MAP kinase‐interacting protein MoSmi1 regulates development and pathogenicity in Magnaporthe oryzae
Source: Mol Plant Pathol. 2024 Jul 21;25(7):e13493. doi: 10.1111/mpp.13493 (PMC11260997; doi:10.1111/mpp.13493)
Supplement: Supplementary file 9 — TableS1 [file MPP-25-e13493-s008.docx]

**Table S1. Putative MoSmi1-interacting proteins identified using IP-MS**

| **Proteins** | | **Putative functions** | **Unique peptides** |
| --- | --- | --- | --- |
| MGG_00183 | STE/STE11 protein kinase | | 1 |
| MGG_00803 | CAMK/CAMKL/AMPK protein kinase | | 2 |
| MGG_01720 | Transcription regulatory protein Swi3 | | 6 |
| **MGG_01822** | **Mitogen-activated protein kinase Hog1 (Osm1)** | | **1** |
| MGG_02656 | protein kinase Rad3 | | 1 |
| MGG_03649 | protein phosphatase 1 regulatory subunit Sds22 | | 1 |
| MGG_03838 | Ser/Thr protein phosphatase | | 9 |
| MGG_04143 | Ras-like protein Rab-6A | | 5 |
| MGG_04476 | Ras-like protein Rab-6A | | 8 |
| MGG_04478 | Fimbrin | | 6 |
| **MGG_04943** | **Mitogen-activated protein kinase Mps1** | | **2** |
| MGG_05203 | Ran-specific GTPase-activating protein 1 | | 5 |
| MGG_05376 | AGC/NDR protein kinase | | 1 |
| MGG_05427 | microtubule-associated protein RP/EB family member 1 | | 4 |
| MGG_06320 | STE/ STE 20 protein kinase | | 6 |
| MGG_08084 | cell wall biogenesis protein phosphatase Ssd1 | | 3 |
| MGG_08643 | PEK/GCN2 protein kinase | | 1 |
| MGG_09912 | CAMK/CAMK1 protein kinase | | 2 |
