# Supplementary material for: A novel MAP kinase‐interacting protein MoSmi1 regulates development and pathogenicity in Magnaporthe oryzae
Source: Mol Plant Pathol. 2024 Jul 21;25(7):e13493. doi: 10.1111/mpp.13493 (PMC11260997; doi:10.1111/mpp.13493)
Supplement: Supplementary file 11 — TableS3 [file MPP-25-e13493-s011.docx]

**Table S3. Verification of transcriptome analysis by qRT-PCR analysis with randomly selected DEGs.**

| **Transcriptome** | | | | **qRT-PCR** | |
| --- | --- | --- | --- | --- | --- |
| Gene | log_2_(fc) | *P* Value | FDR | Fold change | *P* Value |
| *MGG_15990* | 4.567325 | 1.33E-211 | 1.44E-207 | 40.264218 | 1.93E-05 |
| *MGG_02001* | 3.711373 | 1.07E-125 | 2.89E-122 | 18.044768 | 0.00354 |
| *MGG_00710* | 1.679259 | 3.02E-71 | 2.97E-68 | 3.326719 | 2.58E-05 |
| *MGG_07606* | -2.702435 | 7.59E-71 | 6.84E-68 | 0.171928 | 2.23E-09 |
| *MGG_00930* | 1.616872 | 7.83E-57 | 4.45E-54 | 3.568635 | 3.7E-07 |
| *MGG_10306* | 1.080778 | 1.11E-43 | 4.46E-41 | 3.336251 | 9.55E-06 |
| *MGG_01996* | 4.715328 | 1.82E-43 | 7.01E-41 | 79.894851 | 5.01E-05 |
| *MGG_09728* | 1.200915 | 1.56E-42 | 5.64E-40 | 3.053494 | 2.81E-06 |
| *MGG_12468* | 1.025657 | 7.01E-35 | 1.46E-32 | 1.262057 | 0.000759 |
| *MGG_04213* | -2.636056 | 3.78E-43 | 1.41E-40 | 0.214331 | 4.03E-10 |
| *MGG_01990* | 1.145116 | 3.90E-37 | 9.58E-35 | 3.470680 | 0.000629 |
| *MGG_02006* | 5.357264 | 1.09E-16 | 6.15E-15 | 0.248869 | 2.76E-05 |
| *MGG_06898* | 5.044394 | 7.59E-14 | 3.29E-12 | 89.183109 | 0.004767 |
| *MGG_04674* | -1.140307 | 2.34E-13 | 9.65E-12 | 0.478729 | 3.39E-06 |
| *MGG_13977* | -1.445998 | 0.002575 | 0.014674 | 0.721515 | 0.001721 |
