# Supplementary material for: A novel MAP kinase‐interacting protein MoSmi1 regulates development and pathogenicity in Magnaporthe oryzae
Source: Mol Plant Pathol. 2024 Jul 21;25(7):e13493. doi: 10.1111/mpp.13493 (PMC11260997; doi:10.1111/mpp.13493)
Supplement: Supplementary file 12 — TableS4 [file MPP-25-e13493-s001.docx]

**Table S4 Primers used in this study**

| **Primer name** | **Sequences (5’-3’)** | **Remark** |
| --- | --- | --- |
| MoSmi1_upF | GGTACCCGGGGATCCTCTAGAGCTCTAGCGACCAATGACCGACAA | Amplification of upstream fragment of *MoSMI1* for the gene deletion |
| MoSmi1_upR | TTCATTGTTGACCTCCACTAGCGACTTTTTGCAAGGCCGAC |  |
| MoSmi1_downF | GCAAAGGAATAGAGTAGATAGCGATTCACTCGTCTGAGAGGGC | Amplification of downstream fragment of *MoSMI1* for the gene deletion |
| MoSmi1_downR | ACGACGGCCAGTGCCAAGCTTCCTACCAAAGCGCCCGATGTGT |  |
| MoMps1_upF | GGTACCCGGGGATCCTCTAGATGAGAAGTGTACCCACCGTTGCTG | Amplification of upstream fragment of *MoMPS1* for the gene deletion |
| MoMps1_upR | TTCATTGTTGACCTCCACTAGTTTGGGGTCGTTGGGCTCAA |  |
| MoMps1_downF | GCAAAGGAATAGAGTAGATGGCATGAGATACTGCTGCTGGGCA | Amplification of downstream fragment of *MoMPS1* for the gene deletion |
| MoMps1_downR | ACGACGGCCAGTGCCAAGCTTGCTGACTTGAGCGTATTACGTGATGC |  |
| MoSmi1_P1 | AGACCAAGGTAACATTGACGGGAGG | Amplification of the 5’ upstream fragment using for PCR to confirm the deletion of *MoSMI1* |
| MoSmi1_P2 | CTTCAAAGGGCAAACAAGCTCACG |  |
| MoSmi1_P3 | CACCGCCTGGACGACTAAACCA |  |
| MoMps1_P1 | GGATACGATACCTATACCTTACCACAC | Amplification of the 5’ upstream fragment using for PCR to confirm the deletion of *MoMPS1* |
| MoMps1_P2 | GAACACCTTGAATATCTTGCGG |  |
| MoMps1_P3 | CACCGCCTGGACGACTAAACCA |  |
| MoMps1_P4 | GACACCACGACACTCAAAGTCCTTCAC | Amplification of the 3’ downstream fragment using for PCR to confirm the deletion of *MoMPS1* |
| MoMps1_P5 | GCTGGCGGTTTGGATCAGAGGA |  |
| MoMps1_P6 | CCAGCACTCGTCCGAGGGCAAA |  |
| MoSmi_RT_F | TCTTCACCCTACATAGAAG | Amplification of the fragment using for RT-PCR to confirm the deletion of *MoSMI1* |
| MoSmi1_RT_R | CTCGTGGTTTACCTTTCTCC |  |
| Actin_RT_F | CCAGCCTTCAGTCCTGGGTC | Amplification of the fragment using for RT-PCR as an internal parameter |
| Actin_RT_R | AGGGCAGTGATCTCCTTCTG |  |
| MoSmi1_TZ_F | TAGCCTCGACATTTCCCCGC | Amplification of the fragment using for southern blot to confirm the deletion of *MoSMI1* |
| MoSmi1_TZ_R | ACACTATTGAGCCATTGCGG |  |
| MoSmi1_pYF11_F | ACTCACTATAGGGCGAATTGGGTACTCAAATTGGTTCTGAATTGCACGGTGCGGTGAGGT | Amplification of the native promoter region and full length ORF of *MoSMI1* for fusion with GFP tag |
| MoSmi1_pYF11_R | CACCACCCCGGTGAACAGCTCCTCGCCCTTGCCACGATATCGATCGTCTTCATGGCC |  |
| pFGL820_Histone 1_F | ATTATTATGGAGAAACTCGAGTGCGAAGAAACGTGAGAAGGC | Amplification of the native promoter region and full length ORF of *Histone 1* for fusion with RFP tag |
| pFGL820_Histone 1_R | CTCGCCCTTGCTCACGGTACCTGCGGCGGGTGCCTCGGC |  |
| pFGL820_β-tubulin_F | ATTATTATGGAGAAACTCGAGTTAGTCGGCAAGGATGGAGAGT | Amplification of the native promoter region and full length ORF of *β-tubulin* for fusion with RFP tag |
| pFGL820_β-tubulin_R | CTCGCCCTTGCTCACGGTACCCTCCTCGCCCTCAAGAGGG |  |
| Sep3_pYF11_F | ACTCACTATAGGGCGAATTGGGTACTCAAATTGGTT GGGTGCATCGGCCCCACT | Amplification of the native promoter region and full length ORF of *MoSEP3* for fusion with GFP tag |
| Sep3_pYF11_R | CACCACCCCGGTGAACAGCTCCTCGCCCTTGCTCACACGGAGTGAGAAACCCTTCCTC |  |
| Sep5_pYF11_F | ACTCACTATAGGGCGAATTGGGTACTCAAATTGGTTCCGACCCCAGATCTCAAATCT | Amplification of the native promoter region and full length ORF of *MoSEP5* for fusion with GFP tag |
| Sep5_pYF11_R | CACCACCCCGGTGAACAGCTCCTCGCCCTTGCTCACGTTGCCGTCTTCCCCGTT |  |
| MoSmi1_AD_IF | GCCATGGAGGCCAGTGAATTCATGTCTAATTCGTTCGGGGGT | Amplify *MoSMI1* cDNA fragment for Y2H assay |
| MoSmi1_AD_IR | CAGCTCGAGCTCGATGGATCCCTAGATATCGATCGTCTTCATGGC |  |
| MoSmi1_AD_IIF | GCCATGGAGGCCAGTGAATTCATGGGCTGCACCAACAACGATCT | Amplify *MoSMI1^ΔN^* cDNA fragment for Y2H assay |
| MoSmi1_AD_IIR | CAGCTCGAGCTCGATGGATCCCTAGATATCGATCGTCTTCATGGC |  |
| MoSmi1_AD_IIIF1 | GCCATGGAGGCCAGTGAATTCATGTCTAATTCGTTCGGGGGT | Amplify *MoSMI1^ΔSMI1_KNR4^* cDNA fragment for Y2H assay |
| MoSmi1_AD_IIIR1 | TACATATCGTCCGCAACCCTCGCAAACCTGGTCGTACAA |  |
| MoSmi1_AD_IIIF2 | AGGGTTGCGGACGATATGTACAGC |  |
| MoSmi1_AD_IIIR2 | CAGCTCGAGCTCGATGGATCCCTAGATATCGATCGTCTTCATGGC |  |
| MoSmi1_AD_IVF | GCCATGGAGGCCAGTGAATTCATGTCTAATTCGTTCGGGGGT | Amplify *MoSMI1^ΔC^* cDNA fragment for Y2H assay |
| MoSmi1_AD_IVR | CAGCTCGAGCTCGATGGATCCCTAGTGGCCAAGAATGCTGACC |  |
| MoSmi1_AD_VF | GCCATGGAGGCCAGTGAATTCATGTCTAATTCGTTCGGGGGT | Amplify *MoSMI1^ΔSMI1_KNR4ΔC^* cDNA fragment for Y2H assay |
| MoSmi1_AD_VR | CAGCTCGAGCTCGATGGATCCCTACTCGCAAACCTGGTCGTACA |  |
| MoSmi1_AD_VIF | GCCATGGAGGCCAGTGAATTCATGGGCTGCACCAACAACG | Amplify *MoSMI1^ΔNΔC^* cDNA fragment for Y2H assay |
| MoSmi1_AD_VIR | CAGCTCGAGCTCGATGGATCCCTAGTGGCCAAGAATGCTGACC |  |
| MoSmi1_AD_VIIF | GCCATGGAGGCCAGTGAATTCATGGGTTGCGGACGATATGT | Amplify *MoSMI1^ΔNΔSMI1_KNR4^* cDNA fragment for Y2H assay |
| MoSmi1_AD_VIIR | CAGCTCGAGCTCGATGGATCCCTAGATATCGATCGTCTTCATGGC |  |
| MoOsm1_BD_F | ATGGCCATGGAGGCCGAATTCATGGCGGAATTCGTGCGG | Amplify *MoOSM1* cDNA fragment for Y2H assay |
| MoOsm1_BD_R | CCGCTGCAGGTCGACGGATCCTTATTGGCCGGTAAACTGGTCG |  |
| MoMps1_BD_F | ATGGCCATGGAGGCCGAATTCATGTCGGATCTCCAGGGCC | Amplify *MoMPS1* cDNA fragment for Y2H assay |
| MoMps1_BD_R | CCGCTGCAGGTCGACGGATCCTCACCTCCTCTGATCCAAACCG |  |
| MoMps1_Flag_F | CAGGAATTCGATATCAAGCTTATGTCGGATCTCCAGGGCC | Amplification of the full length ORF of *MoMPS1* for fusion with Flag tag |
| MoMps1_Flag_R | GTCGACGGTATCGATAAGCTTCCTCCTCTGATCCAAACCGC |  |
| MoOsm1_Flag_F | CAGGAATTCGATATCAAGCTTATGGCGGAATTCGTGCGG | Amplification of the full length ORF of *MoOSM1* for fusion with Flag tag |
| MoOsm1_Flag_R | GTCGACGGTATCGATAAGCTTTTGGCCGGTAAACTGGTCG |  |
| MoSmi1_YFPC_F | CAATCACAATGGCCGGATCCATGTCTAATTCGTGAGCTTGTTT | Amplification of the full length ORF of *MoSMI1* for fusion with C-terminal YFP tag |
| MoSmi1_YFPC_R | CTTGCAGGCCGGGCGCCCGGGGATATCGATCGTCTTCATGGC |  |
| MoMps1_YFPN_F | GTCTATATCATGGCCTCTAGAATGTCGGATCTCCAGGGCCGC | Amplification of the full length ORF of *MoMPS1* for fusion with N-terminal YFP tag |
| MoMps1_YFPN_R | GTCGCTTACTGCAGGTCGACCCTCCTCTGATCCAAACCGCC |  |
| MoOsm1_YFPN_F | GTCTATATCATGGCCTCTAGAATGGCGGAATTCGTGCGGGCC | Amplification of the full length ORF of *MoOSM1* for fusion with N-terminal YFP tag |
| MoOsm1_YFPN_R | GTCGCTTACTGCAGGTCGACTTGGCCGGTAAACTGGTCGTCCA |  |
| β-tubulin_qF | CCAGCCTTCAGTCCTGGGTC | qRT-PCR of *β-tubulin* |
| β-tubulin_qR | AGGGCAGTGATCTCCTTCTG |  |
| MoSmi1_qF | TCTTCACCCTACATAGAAG | qRT-PCR of *MoSMI1* |
| MoSmi1_qR | CTCGTGGTTTACCTTTCTCC |  |
| HPH_qF | ATGTCCTGCGGGTAAATAGC | qRT-PCR of *HPH* |
| HPH_qR | GATGCAATAGGTCAGGCTCTC |  |
| Apx2_qF | CGTCGTCACCGAGTATCTGG | qRT-PCR of *APX2* |
| Apx2_qR | CCTCATTGCGTTGACGGTTG |  |
| Prx1_qF | CTACCAGGACACCACCAACG | qRT-PCR of *PRX1* |
| Prx1_qR | CCGGGTACGACAGAATGGTC |  |
| Atf1_qF | CGGCAAACGGCCTCTTTATG | qRT-PCR of *ATF1* |
| Atf1_qR | AGGTGACGTCTTGATGGCAG |  |
| Hyr1_qF | ATGGCTTCCGCTACGACAAT | qRT-PCR of *HYR1* |
| Hyr1_qR | TTGGAGGCCGTGTTGACTAC |  |
| Tpx1_qF | CTCTGTGAACGGCAAGGAGT | qRT-PCR of *TPX1* |
| Tpx1_qR | ACCAGTTGCTTCAGGGTGAC |  |
| Trx2_qF | TTTCAAATCCGGTCGCTTGC | qRT-PCR of *TRX2* |
| Trx2_qR | TTGTTGCTGATTGCGACGTG |  |
| Ccp1_qF | CCGTGTACAACGACATTGCC | qRT-PCR of *CCP1* |
| Ccp1_qR | CTCCTTGTCGTAGGTACCGC |  |
| Nmo1_qF | AGGAGTCAGGCGACAATTCC | qRT-PCR of *NMO1* |
| Nmo1_qR | CTCTTCTTTGCCTCGTCCCT |  |
| Lhs1_qF | AACCAGCTCGAGGGCTTTAC | qRT-PCR of *LHS1* |
| Lhs1_qR | TTTCTCGAGCTTTGTCCGCT |  |
| Kar2_qF | TGGGCAAGAAGGTTACCCAC | qRT-PCR of *KAR2* |
| Kar2_qR | AGAACGTTGAGACCGGCAAT |  |
| MGG_15990_qF | TTTGTGTTCCTCTTTGCCGC | qRT-PCR of 15 DEGs |
| MGG_15990_qR | CGAGGGTCAGGGATCCTGCA |  |
| MGG_02001_qF | TTCATCTGCCGCTTCTACGC |  |
| MGG_02001_qR | GCGCCAGGATCTGCGGGATC |  |
| MGG_00710_qF | ACGATCATCCTTTTCGGTGC |  |
| MGG_00710_qR | GAAGTCATCGTTCGAGGGCA |  |
| MGG_07606_qF | ATATGCCACTGGATGGAACT |  |
| MGG_07606_qR | GGCCATACCCAGCACAATGA |  |
| MGG_00930_qF | CATTGTGACGGCTGTTGTTA |  |
| MGG_00930_qR | CAATGGCAACAGTGGCCCTC |  |
| MGG_10306_qF | ATGTCCCAAAGGGCTTACGA |  |
| MGG_10306_qR | GCTGCATATATCCTGGCCAT |  |
| MGG_01996_qF | TTCCTCAGCTCAGCTGCAAC |  |
| MGG_01996_qR | TGGTCTGGGCTTTGGGAATA |  |
| MGG_09728_qF | TTTTACAACTGTGGCTGGTT |  |
| MGG_09728_qR | CGACAGGACCGAGTTGAGCA |  |
| MGG_12468_qF | AACAGCAGTCCACGCTGCAT |  |
| MGG_12468_qR | GAAATGATCACCAGCGTACG |  |
| MGG_04213_qF | GAAAGGCCATGTAGGCAATG |  |
| MGG_04213_qR | ACTCAGCTTGACCGACATCT |  |
| MGG_01990_qF | GTCGCCGCAACCTCTGATCA |  |
| MGG_01990_qR | TCTTGCCTCGCAAGCTTGAG |  |
| MGG_02006_qF | ATTGGAAACGCTGGCACTGG |  |
| MGG_02006_qR | TGCAACCCTGTTCCGCTCAA |  |
| MGG_06898_qF | CCTGCCTCCAATTGTTACAG |  |
| MGG_06898_qR | CTAGGCACGTCGGGCATGCG |  |
| MGG_04674_qF | GGAACAAACGATTTCGTATT |  |
| MGG_04674_qR | GTGATGAACTTGTACGACCG |  |
| MGG_13977_qF | GTCGGCGCCCAAGCGCTTCA |  |
| MGG_13977_qR | CGATGATACCAGCAACATGC |  |
